# 5-Aminolevulinic Acid: A Novel Approach to Improving Radioresistance in Prostate Cancer

**DOI:** 10.3390/cancers17081286

**Published:** 2025-04-10

**Authors:** Fumisato Maesaka, Yasushi Nakai, Takanori Yoshida, Mitsuru Tomizawa, Takuto Shimizu, Takuya Owari, Kenta Onishi, Makito Miyake, Hiroki Kuniyasu, Kiyohide Fujimoto, Nobumichi Tanaka

**Affiliations:** 1 Department of Prostate Brachytherapy, Nara Medical University, 840 Shijo-cho, Kashihara-shi 634-8522, Nara, Japan; mae_fumi0107@yahoo.co.jp (F.M.); nakaiyasusiuro@live.jp (Y.N.); kote.men.dooooh@gmail.com (T.Y.); 2 Department of Urology, Nara Medical University, 840 Shijo-cho, Kashihara-shi 634-8522, Nara, Japan; tomimit.com@gmail.com (M.T.); takutea19@gmail.com (T.S.); tintherye@gmail.com (T.O.); kenzmedico0912@yahoo.co.jp (K.O.); makitomiyake@yahoo.co.jp (M.M.); kiyokun@naramed-u.ac.jp (K.F.); 3 Department of Molecular Pathology, Nara Medical University, 840 Shijo-cho, Kashihara-shi 634-8522, Nara, Japan; cooninh@zb4.so-net.ne.jp

**Keywords:** prostate cancer, radioresistance, 5-aminolevulinic acid, protoporphyrin IX, ABCG2

## Abstract

**Background/Objectives**: Prostate cancer (PCa) cells may acquire radioresistance during radiation therapy (RT), resulting in PCa recurrence. This study was aimed at investigating the radiosensitizing effect of 5-aminolevulinic acid (5-ALA) on radioresistant PCa cells. **Methods**: Radioresistant PCa cells were developed through successive irradiation of two human PCa cell lines (PC-3 and DU 145) and a murine PCa cell line (Myc-CaP). The radiosensitivity of these PCa cells and the radiosensitizing effect of 5-ALA were evaluated using clonogenic assays. Mitochondrial accumulation of protoporphyrin IX (PpIX) and mitochondrial reactive oxygen species (ROS) were evaluated. A syngeneic mouse model with radioresistant PCa was established, and the immunohistochemistry of cell specimens from PCa patients with local recurrence after primary RT was examined. **Results**: Radioresistant PCa cells showed lower radiosensitivity compared to parental PCa cells. In radioresistant PCa cells with 5-ALA administration, compared to the group administered irradiation alone, the survival rate after irradiation was significantly reduced by promoting mitochondria-mediated apoptosis caused by increased PpIX accumulation and mitochondrial ROS generation. Similar results were observed in vivo. However, compared with parental PCa cells, radioresistant PCa cells were less affected by the radiosensitizing effect of 5-ALA, owing to decreased PpIX accumulation and mitochondrial ROS production caused by upregulated expression of the drug transporter ABCG2. ABCG2 expression was upregulated in human PCa specimens with post-RT recurrence. **Conclusions**: 5-ALA enhanced the antitumor effects of RT in radioresistant PCa cells; however, ABCG2 upregulation decreased PpIX accumulation, resulting in a reduced radiosensitizing effect of 5-ALA on radioresistant PCa cells compared with that on parental PCa cells. ABCG2 could be a potential therapeutic target for overcoming radioresistance.

## Simple Summary

This study aimed to investigate the radiosensitizing effect of 5-aminolevulinic acid on radioresistant prostate cancer cells. Radioresistant prostate cancer cells with low radiosensitivity were developed by consecutively irradiating prostate cancer cell lines. In radioresistant prostate cancer cells, combination therapy of irradiation with 5-aminolevulinic acid increased radiosensitivity by promoting apoptosis, mediated by mitochondria, via increased accumulation of protoporphyrin IX and mitochondrial reactive oxygen species. However, upregulation of the drug transporter ABCG2 in radioresistant cells reduced the accumulation of protoporphyrin IX and mitochondrial reactive oxygen species, thereby diminishing the radiosensitizing effect. Moreover, ABCG2 expression was higher in specimens from patients with prostate cancer that recurred post-radiation. The study suggests that 5-aminolevulinic acid could improve radioresistance, with ABCG2 as a potential therapeutic target.

## 1. Introduction

The global incidence of prostate cancer (PCa) is rising due to the widespread use of prostate-specific antigens, multiparametric magnetic resonance imaging, and the Prostate Health Index, with an estimated 1.41 million cases diagnosed in 2020 [1,2]. Although various treatment options for PCa exist, including surgery, hormonal therapy, and chemotherapy [3], radiation therapy (RT) serves as a pivotal radical treatment for localized PCa, particularly among patients who are ineligible for surgery [4,5]. Moreover, combining RT with androgen deprivation therapy has shown improved oncological outcomes compared to surgery [5,6,7]. Nevertheless, biochemical recurrence affects 25–48% of high-risk cases with elevated tumor grades [7,8,9]. Salvage RT is one of the treatment options for patients with locally recurrent PCa after primary RT, which occurs in approximately 3–10% of patients following radiation therapy [10,11]. However, due to its limited oncological outcomes and the increase in the occurrence of adverse events, an established standard treatment has not yet been determined [12]. The limited oncological outcomes of salvage RT can be attributed to the acquisition of radioresistance in recurrent PCa cells following RT [13]. Mechanisms such as reduced levels of reactive oxygen species (ROS) due to tumor hypoxia [14,15], acquisition of stemness [16], enhanced DNA repair, and apoptosis suppression [17,18] have been implicated in radioresistance. By adopting a novel approach targeting these mechanisms, it is expected to improve the oncological outcomes of salvage RT and suppress adverse events by reducing the radiation dose.

5-Aminolevulinic acid (5-ALA) is a natural amino acid and an FDA-approved drug that is converted to protoporphyrin IX (PpIX) within the mitochondria in normal cells, through the heme synthesis pathway, and is ultimately metabolized into heme [19]. However, in cancer cells, the demand for heme and the supply of Fe^2+^ are reduced due to the Warburg effect. As a result, exogenous 5-ALA administration leads to the excessive accumulation of PpIX in cancer cells [20]. PpIX generates mitochondrial ROS when excited by specific wavelengths of 400–410 nm, and it is used in photodynamic therapy (PDT) for cancer [20,21]. Furthermore, the antitumor effects of radiodynamic therapy combining 5-ALA and RT have been reported for tumors such as glioblastoma and lymphoma, where visible-light irradiation is not feasible [22,23]. Our previous research demonstrated the radiosensitizing effect of 5-ALA through radiodynamic therapy in PCa cells [24,25]. The mechanism underlying the radiosensitizing effect of 5-ALA involves the excitation of PpIX, which accumulates in the mitochondria of PCa cells, similar to PDT [24,25]. This radiation-induced excitation leads to increased mitochondrial ROS production, resulting in mitochondrial dysfunction and induction of mitochondria-mediated apoptosis [24]. Furthermore, 5-ALA was shown to enhance the antitumor effects of RT even under hypoxic conditions with low radiosensitivity [24]. Recent studies have also reported the enhancement of the cytotoxic effects of a combination therapy involving 5-ALA and laser irradiation against radioresistant esophageal cancer [26].

In the present study, we aimed to evaluate the radiosensitizing effects of 5-ALA in radioresistant PCa cells under hypoxic conditions, as well as to explore a new approach to increasing the radiosensitivity of radioresistant PCa cells. Ultimately, the goal was to demonstrate the potential utility of 5-ALA as an adjuvant therapy in salvage RT for PCa with local recurrence following primary RT. To achieve these objectives, we developed radioresistant PCa cells and assessed PpIX accumulation, mitochondrial ROS, and apoptosis in these cells. Additionally, we analyzed the immunohistochemistry of human specimens of PCa that recurred after primary RT, which may help in developing new therapeutic strategies for overcoming radioresistance in the future.

## 2. Materials and Methods

### 2.1. Cell Lines and Culture Conditions

Human PCa cell lines, including PC-3 established from bone metastases [27] and DU 145 established from brain metastases [28], along with Myc-Cap derived from murine PCa [29], were purchased from the American Type Culture Collection (Manassas, VA, USA). Cells were cultured in RPMI 1640 growth medium (Nissui, Tokyo, Japan) supplemented with 10% FBS (ICN Biomedicals, Aurora, OH, USA), 100 U/mL penicillin, and 100 µg/mL streptomycin (Gibco, Grand Island, NY, USA) in a standard humidified incubator at 37 °C and 5% CO_2_.

### 2.2. Reagents and Equipment

The 5-ALA was purchased from SBI Pharmaceuticals Co. Ltd. (Tokyo, Japan). X-ray irradiation (IR) was performed using a 150 kVp X-ray generator (Model MBR-1520R; Hitachi, Tokyo, Japan). Absorbance was measured in a cell viability assay, and PpIX was measured using a microplate spectrophotometer (Infinite 200 M PRO, Tecan, Männedorf, Switzerland) equipped with i-control software (version 1.8). The Moxi GOTM II (Orflo Technologies, Ketchum, ID, USA) was the cell analyzer used. The flow cytometry data were analyzed using FlowJoTM v10 (BD Biosciences, Bedford, MA, USA). Microscopy was conducted using an EVOS FL Auto (Thermo Fisher Scientific, Waltham, MA, USA), which integrates the functionalities of both optical and fluorescence microscopes.

### 2.3. Cytotoxicity of 5-ALA

Based on our previous study [24], the concentration of 5-ALA administered in this experiment was set at 1 mM. To evaluate the validity of this concentration, the cytotoxicity of 5-ALA was assessed by seeding 1 × 10^3^ cells per well in a 96-well plate, adding 5-ALA to the wells at various concentrations (control, 0.01 mM, 0.1 mM, 0.5 mM, 1 mM, 2 mM, 5 mM, or 10 mM) on the following day, and measuring the absorbance 24 h later at 490 nm, with reference absorbance at 630 nm, using a microplate spectrophotometer and a cell counting kit-8 (Dojindo Laboratories, Inc., Kumamoto, Japan) according to the manufacturer’s protocol. Cell viability was calculated based on the absorbance values obtained, and the half-maximal inhibitory concentration (IC_50_) was calculated from a sigmoidal curve using a nonlinear regression model generated using GraphPad Prism 9.5.1 (GraphPad Software, San Diego, CA, USA).

### 2.4. Assessment of Radiosensitivity

The cell survival rates after IR were estimated using a clonogenic assay, as described in our previous report [25]. Since PpIX accumulation peaks at 3–6 h after 5-ALA administration [30,31], IR was performed 3 h after 5-ALA administration in this experiment. Cells (1–2 × 10^3^) were seeded in 12- or 24-well plates in the same amount as the corresponding controls, followed by IR (2, 4, 6, or 8 Gy single dose) 24 h later. For the groups treated with IR and 5-ALA, 1 mM 5-ALA was administered in the dark 3 h before IR, and post-treatment cultures were maintained in the dark. The cells from all groups were then cultured for 7–10 days, fixed with 1% glutaraldehyde, stained with crystal violet, and counted. Viable colonies were defined as those containing more than 50 cells. Based on the number of viable colonies in each group, the relative cell survival rate at each dose of IR was calculated from the control, and a linear–quadratic model [32] was constructed using GraphPad Prism 9.5.1 (GraphPad Software). Furthermore, the radiation dose (D_50%_) required to achieve a 50% survival rate after IR was calculated from the linear–quadratic curve.

### 2.5. Establishment of Radioresistant PCa Cells

PCa cells (PC-3, DU 145, and Myc-CaP) were cultured in RPMI 1640 medium containing FBS, penicillin, and streptomycin. Following the protocol described by Kuwahara et al. in several studies [33,34,35], the cells were irradiated daily at 0.5 Gy using an X-ray generator for 30 consecutive days. When cell proliferation was observed, the radiation dose was gradually increased to 1 Gy × 30 days, 1.5 Gy × 30 days, and 2 Gy × 30 days. If a halt in cell proliferation was observed during treatment, the radiation dose was reduced, or IR was temporarily discontinued for a few days. Cells that continued to proliferate under daily 2 Gy IR were defined as radioresistant PCa cells (PC-3-R, DU 145-R, and Myc-CaP-R).

### 2.6. Apoptosis Analysis

In total, 1.5 × 10^5^ cells were seeded in four wells in a six-well plate and then assigned to one of the following groups for treatment after 24 h: normal control, 5-ALA alone, IR alone, and 5-ALA combined with IR. In the IR-alone group, a single dose of 4 Gy was administered. In the 5-ALA combined with IR group, 1 mM 5-ALA was added 3 h prior to IR under dark conditions, and the samples were maintained in light-protected conditions until analysis. Twenty-four hours after the treatment, the cells from all groups were processed using the ApoFlowEx FITC Kit (exbio, Nad Safinou, Czech Republic) according to the manufacturer’s protocol and analyzed using the Moxi GO™ II (Orflo Technologies) at a cell count of 2–5 × 10^4^. The flow cytometry data were further analyzed using FlowJo™ v10 software (BD Biosciences), and the proportion of early apoptosis was compared among the groups.

### 2.7. Western Blot Analysis

Proteins were extracted from whole cells using a radioimmunoprecipitation assay buffer (Sigma-Aldrich; Merck KGaA, Darmstadt, Germany) and a protein inhibitor cocktail (Nacalai Tesque, Kyoto, Japan), according to the manufacturer’s protocol. In the treatment groups, the cells were extracted 24 h after treatment. The protein concentrations were measured using a Protein Assay BCA kit (Nacalai Tesque, Inc.), and Western blot analysis was performed as described in our previous report [36]. The extracted proteins were diluted in sodium dodecyl sulfate loading buffer containing 2.5% β-mercaptoethanol and heated at 95 °C for 5 min. Subsequently, an equal amount of protein (10 μg) was loaded into each well of a 10% sodium dodecyl sulfate–polyacrylamide gel. Electrophoresis was performed at 200 V for 35 min using a Mini-Protean Tetra Cell (Bio-Rad Laboratories, Inc., Hercules, CA, USA). The proteins were then transferred onto polyvinylidene difluoride membranes (Hybond-P; GE Healthcare, Chicago, IL, USA; Cytiva, Marlborough, MA, USA) using a semidry transfer apparatus (Trans-Blot SD Semi-Dry Transfer Cell; Bio-Rad Laboratories, Inc.) at 15 V for 45 min. After blocking with Tris-buffered saline (pH 7.6) containing 5% skimmed milk for 1 h at room temperature, the membrane was incubated overnight at 4 °C with primary antibodies, followed by incubation with secondary antibodies for 1 h at room temperature. The primary antibodies used included anti-β-actin rabbit polyclonal antibody (cat. no. 20536-1-AP; dilution 1:2000; Proteintech, Rosemont, IL, USA), anti-hypoxia-inducible factor 1a (HIF-1a) rabbit polyclonal antibody (cat. No. R12-2180; dilution 1:500; Assay Biotechnology, Fremont, CA, USA), anti-HIF-2a rabbit polyclonal antibody (cat. no. NB100-122; dilution 1:500; Novus Biologicals, Centennial, CO, USA), anti-ATP-binding cassette transporter subfamily G2 (ABCG2) rabbit polyclonal antibody (cat. no. 27286-1-AP; dilution 1:1000; Proteintech), anti-ferrochelatase murine monoclonal antibody (cat. no. sc-377377; dilution 1:500; Santa Cruz Biotechnology, Dallas, TX, USA), anti-proton-coupled peptide transporter 1 (PEPT-1) rabbit polyclonal antibody (cat. no. sc-20653; dilution 1:200; Proteintech), anti-B-cell/CLL lymphoma 2 (BCL-2) murine monoclonal antibody (cat. no. sc-7382; dilution, 1:200; Santa Cruz Bio-technology), anti-BCL-2-associated X protein (BAX) rabbit polyclonal antibody (cat. no. sc-526; dilution, 1:200; Santa Cruz Biotechnology), anti-BCL-2-associated agonist of cell death (BAD) murine monoclonal antibody (cat. no. sc-8044; dilution 1:200; Santa Cruz Biotechnology), and anti-BCL extra-large (BCL-xL) murine monoclonal antibody (cat. no. sc-8392; dilution 1:200; Santa Cruz Biotechnology). The secondary antibodies were horseradish peroxidase-conjugated goat anti-mouse IgG (cat. no. SA00001 1; dilution 1:10,000; Proteintech) or anti-rabbit IgG antibody (cat. no. SA00001 2; dilution 1:10,000; Proteintech). Finally, the bands were observed by detecting secondary antibodies using SuperSignal West Pico Chemiluminescent Substrate (Thermo Fisher Scientific). The detected bands were quantified using the ImageJ software (version 1.52). Protein levels were calculated based on the protein quantity of β-actin as a reference, and the experiment was performed thrice.

### 2.8. Evaluation of Mitochondrial ROS

Post-treatment mitochondrial ROS (mitochondrial superoxide) were visualized using MitoSOX Red (Thermo Fisher Scientific), following the manufacturer’s protocol, and measurements were performed 1, 6, and 12 h after IR. Subsequently, the cells were observed under a fluorescence microscope using an RFP light cube (excitation: 531 nm, emission: 593 nm). The 5-ALA combined with the IR group received 5-ALA in darkness 3 h before IR (4 Gy single dose) and was shielded from light until the measurements were performed. Fluorescence intensity was measured by selecting the 20 cells with the highest fluorescence intensity from images captured using a fluorescence microscope. The selection was performed by three independent researchers. The intensity was quantified using the ImageJ software (version 1.52) and compared among the groups.

### 2.9. Quantification of Intracellular PpIX Expression

Intracellular PpIX expression was quantified using a microplate spectrophotometer. Measurements were taken in the 5-ALA-treated group 3 h after the administration of 1 mM 5-ALA. Based on our previous report [37], the measurement method included excitation with light at a wavelength of 400 nm, and the fluorescence intensity at 630–640 nm was quantified as the level of PpIX expression.

### 2.10. Transfection of Small Interfering RNA (siRNA) ABCG2

PCa cells seeded at 5 × 10^5^ cells/well in 6-well plates were transfected with synthesized siRNA ABCG2 (si ABCG2; cat. no. 118156; Invitrogen, Waltham, MA, USA; Life Technologies, Carlsbad, CA, USA; Thermo Fisher Scientific, Tokyo, Japan) or siRNA negative control (si NC; cat. no. 4390843; Invitrogen; Life Technologies; Thermo Fisher Scientific). First, the cells were incubated with 50 pmol of siRNA and 5 µL of Lipofectamine 2000 (Life Technologies; Thermo Fisher Scientific) according to the manufacturer’s instructions at 37 °C for 48 h. Following transfection, ABCG2 expression was measured by Western blot analysis.

### 2.11. Syngeneic Mouse Model with Radioresistant PCa

This animal study was approved by the Committee on Animal Research of Nara Medical University (approval number: 13460, 2 March 2023). All animal experiments were conducted in accordance with the Guidelines for Welfare of Animals in Experimental Neoplasia. Following our previous report [24], we used FVB/NJcl mice (5 weeks old, male; CLEA Japan, Tokyo, Japan). For the subcutaneous tumor model, syngeneic Myc-CaP-R cells (1 × 10^5^ cells) in Matrigel (BD Biosciences, San Jose, CA, USA) were subcutaneously inoculated into the thigh. The treatments were initiated on day 14 when the tumors reached 1000 mm^3^. The mice were divided into four groups: normal control, 5-ALA alone, IR alone, and IR with 5-ALA. While we cannot entirely rule out the possibility that the number of animals may have been insufficient, this study was conducted with 12 mice in total, with 3 mice per group, in adherence to the 3R rule. The mice were subjected to a radiation dose of 2 Gy per fraction (1 Gy/min) for 10 days, with their bodies protected using a lead collimator. In the IR with 5-ALA group, 5-ALA (30 mg/kg) was orally administered 3 h before each application of IR in the dark. These procedures were performed under anesthesia with 1.0–2.0% isoflurane. Body weight and tumor size were measured three times per week. Humane endpoints were defined by total tumor volume > 10% of body weight, tumor diameter > 20 mm, weight loss > 20%, tumor ulceration, necrosis, gait disturbance, and impaired water and food intake. Tumor volume (mm^3^) was calculated using the following formula: length (mm) × width (mm) × height (mm) × 0.52. The mice were euthanized 7 days after the last radiation fraction via cervical dislocation, under inhalation anesthesia using 2.0–2.5% isoflurane, and their tumors were excised.

### 2.12. Immunohistochemistry

Human specimens and tumors collected from mice were fixed in 10% neutral-buffered formalin at room temperature, followed by the creation of paraffin blocks. Immunohistochemical staining was performed as described in our previous report [36]. The primary antibodies used included anti-HIF-1a rabbit polyclonal antibody (cat. no. R12-2180; dilution 1:100; Assay Biotechnology Company, Fremont, CA, USA), anti-ABCG2 rabbit polyclonal antibody (cat. no. 27286-1-AP; dilution 1:100; Proteintech), anti-4-hydroxynonenal (4-HNE) rabbit polyclonal antibody (cat. no. ab46545; dilution 1:200; Abcam, Cambridge, UK), and anti-cleaved caspase 3 (C-caspase 3) rabbit monoclonal antibody (cat. no. 9664; dilution 1:200; Cell Signaling Technology, Danvers, MA, USA). The secondary anti-mouse and anti-rabbit IgG antibodies were included in the Histofine SAB PO kit (Nichirei Biosciences, Inc., Tokyo, Japan) at 100 µL (dilution 1:1000). Finally, observations were made under an optical microscope at magnifications ranging from 200× to 400×.

Immunohistochemical positivity was evaluated using the Allred scoring system, which is widely used to diagnose breast cancer [38]. The Allred score combines the proportion and intensity scores, resulting in a total score ranging from 0 to 8. The estimated proportion of positively stained tumor cells was classified into six categories (0–5), and the average intensity of positively stained tumor cells (0–3) was categorized into four levels. Additionally, scoring was conducted in a blinded manner by three independent researchers.

### 2.13. Patients

Biopsy specimens from 15 patients with local recurrence in the prostate or seminal vesicles at Nara Medical University Hospital between April 2011 and December 2020, after treatment with radical RT (external beam IR and/or low-dose-rate brachytherapy) for localized PCa, were included in this study. Additionally, biopsy specimens from 7 patients with available pre-RT prostate biopsy samples among the 15 cases were also included. The Nara Medical University Hospital Institutional Review Board approved this study, and informed consent was obtained from all patients (approval number: 3622). This retrospective study used existing specimens and did not involve any interventions. Therefore, an opt-out document was displayed in the examination room of our hospital to ensure that the patients were given the opportunity to decline participation in this study.

### 2.14. Statistical Analysis

GraphPad Prism 9.5.1 (GraphPad Software) was used for the statistical analysis. The comparisons of IC_50_ values and survival curves after IR between the radioresistant and parental PCa cells were performed using the extra sum-of-squares F test. Group comparisons were conducted using two-tailed Student’s *t*-tests or one-way ANOVA when there was a single variable and two-way ANOVA when there were two or more variables. Post hoc multiple comparisons between groups were performed using Tukey’s multiple comparison test or Sidak’s multiple comparison test, resulting in the calculation of adjusted *p*-values. Statistical significance was set at *p* < 0.05.

## 3. Results

### 3.1. Cytotoxicity of 5-ALA Against Parental and Radioresistant PCa Cells

**Figure 1:**
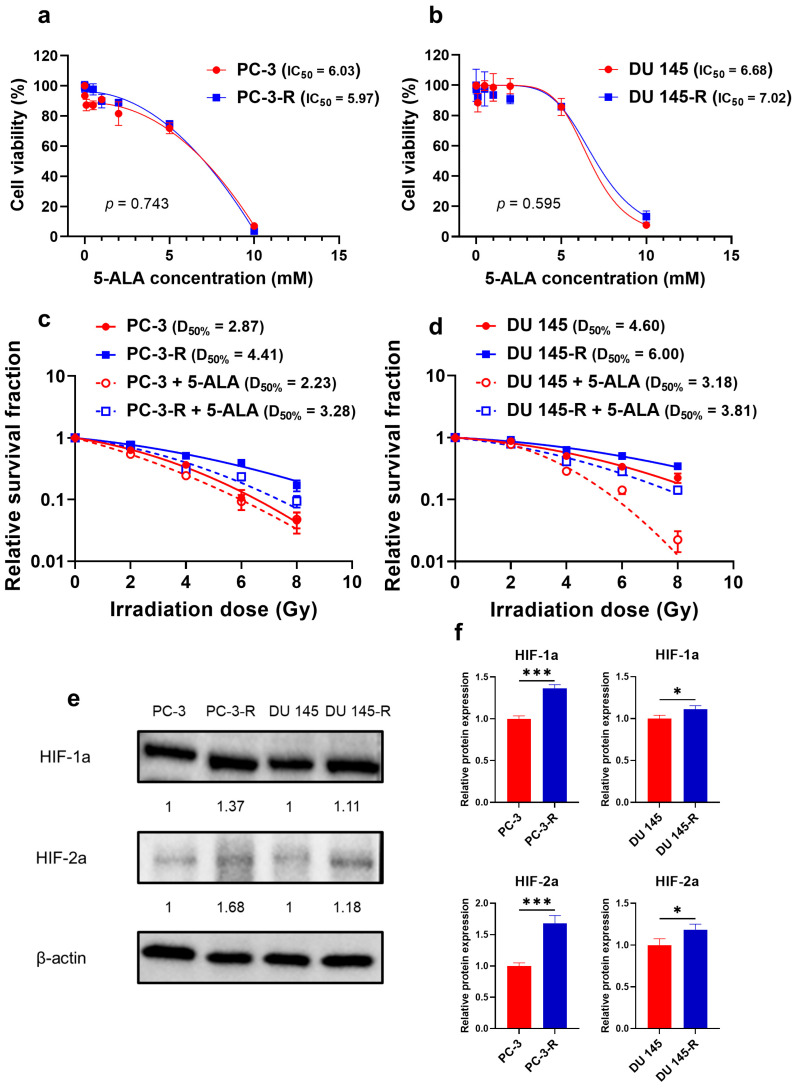
The viability of parental PCa cells and the established radioresistant PCa cells 24 h after 5-ALA administration ((**a**) PC-3 and PC-3-R; (**b**) DU 145 and DU 145-R). The survival curves of the relative survival fractions in the parental PCa cells and the established radioresistant PCa cells after IR (2–8 Gy single dose) alone or a combination of 1 mM 5-ALA and IR ((**c**) PC-3 and PC-3-R; (**d**) DU 145 and DU 145-R). (**e**,**f**) HIF-1a and HIF-2a expression in the parental and radioresistant PCa cells was measured via Western blot analysis. 5-ALA: 5-aminolevurinic acid. D_50%_: radiation dose required to achieve a 50% survival rate. HIF: hypoxia-inducible factor. IC_50_: half-maximal inhibitory concentration. IR: irradiation. PCa: prostate cancer. * *p* < 0.05, *** *p* < 0.001. Original western blots are presented in File S1.

The parental and radioresistant PCa cells exhibited the cytotoxic effects of 5-ALA treatment. The IC_50_ values were 6.03 mM and 5.97 mM for PC-3 and PC-3-R cells, respectively (Figure 1a; *p* = 0.743), while they were 6.68 mM and 7.02 mM for DU 145 and DU 145-R cells, respectively (Figure 1b; *p* = 0.595). The cell viability following the administration of 1 mM 5-ALA was 90.9 ± 1.6%, 89.9 ± 3.7%, 98.6 ± 7.5%, and 93.6 ± 3.4% for PC-3, PC-3-R, DU 145, and DU 145-R cells, respectively.

### 3.2. Establishment of Radioresistant PCa Cells

A representative image depicting the clonogenic assay results is shown in Figure S1a. The survival curves of the established radioresistant PCa cell lines (PC-3-R and DU 145-R) after IR were compared with those of the parental PCa cell lines (PC-3 and DU 145). PC-3-R cells exhibited significantly reduced radiosensitivity compared to PC-3 cells (*p* < 0.001; PC-3, A value = 0.156, B value = 0.030, D_50%_ = 2.87 Gy; PC-3-R, A value = 0.101, B value = 0.013, D_50%_ = 4.41 Gy; Figure 1c). DU 145-R cells exhibited significantly reduced radiosensitivity compared to DU 145 cells (*p* < 0.001; DU 145, A value = 0.067, B value = 0.018, D_50%_ = 4.60 Gy; DU 145-R, A value = 0.049, B value = 0.011, D_50%_ = 6.00 Gy; Figure 1d). These findings demonstrate that PC-3-R and DU 145-R cells acquired radioresistance compared to the parental PCa cells.

The expression of the hypoxic environment markers HIF-1a and HIF-2a was evaluated via Western blot analysis in the parental and radioresistant cells (Figure 1e,f). In the radioresistant PCa cells, HIF-1a expression was significantly upregulated compared to the parental PCa cells (PC-3-R, *p* < 0.001; DU 145-R, *p* = 0.030). Furthermore, HIF-2a expression was also significantly upregulated in the radioresistant PCa cells (PC-3-R, *p* < 0.001; DU 145-R, *p* = 0.034). These findings indicated that the radioresistant PCa cells form a hypoxic signaling environment compared to the parental PCa cells.

### 3.3. Radiosensitizing Effect of 5-ALA on Parental and Radioresistant PCa Cells

The radiosensitivity of the parental and radioresistant PCa cells treated with IR alone (control treatment) or a combination of 5-ALA and IR was evaluated. In PC-3 cells (Figure 1c), the combination of 5-ALA with IR significantly increased their radiosensitivity (*p* < 0.001; combination of 5-ALA and IR, A value = 0.267, B value = 0.020, D_50%_ = 2.23 Gy). In DU 145 cells (Figure 1d), radiosensitivity was significantly improved following treatment with the combination of 5-ALA and IR (*p* < 0.001; combination of 5-ALA and IR, A value = 0.001, B value = 0.068, D_50%_ = 3.18 Gy).

Similarly, in PC-3-R cells (Figure 1c), the combination of 5-ALA and IR significantly increased their radiosensitivity (*p* < 0.001; combination of 5-ALA and IR, A value = 0.129, B value = 0.025, D_50%_ = 3.28 Gy). In DU 145-R cells (Figure 1d), it was demonstrated that their radiosensitivity significantly increased with the combination of 5-ALA and IR (*p* < 0.001; combination of 5-ALA and IR, A value = 0.109, B value = 0.019, D_50%_ = 3.81 Gy). These findings indicate that 5-ALA exhibits radiosensitizing effects not only on parental but also on radioresistant PCa cells.

### 3.4. Effects of 5-ALA on Apoptosis After IR in Parental and Radioresistant PCa Cells

In the parental PCa cells (PC-3 and DU 145), the early apoptosis rates 24 h after exposure to IR alone or the combination of IR and 5-ALA were assessed using annexin V-FITC and propidium iodide staining via flow cytometry. In PC-3 cells (Figure S2a,b), a significant increase in the early apoptosis rates was observed in the 5-ALA combined with the IR group (IR alone, 12.1 ± 0.7%; 5-ALA + IR, 17.4 ± 1.11%; *p* = 0.004). Similarly, in DU 145 cells (Figure S2c,d), the 5-ALA combined with the IR group also exhibited a significant increase in early apoptosis rates (IR alone, 9.2 ± 0.2%; 5-ALA + IR, 11.2 ± 0.4%; *p* = 0.003).

**Figure 2:**
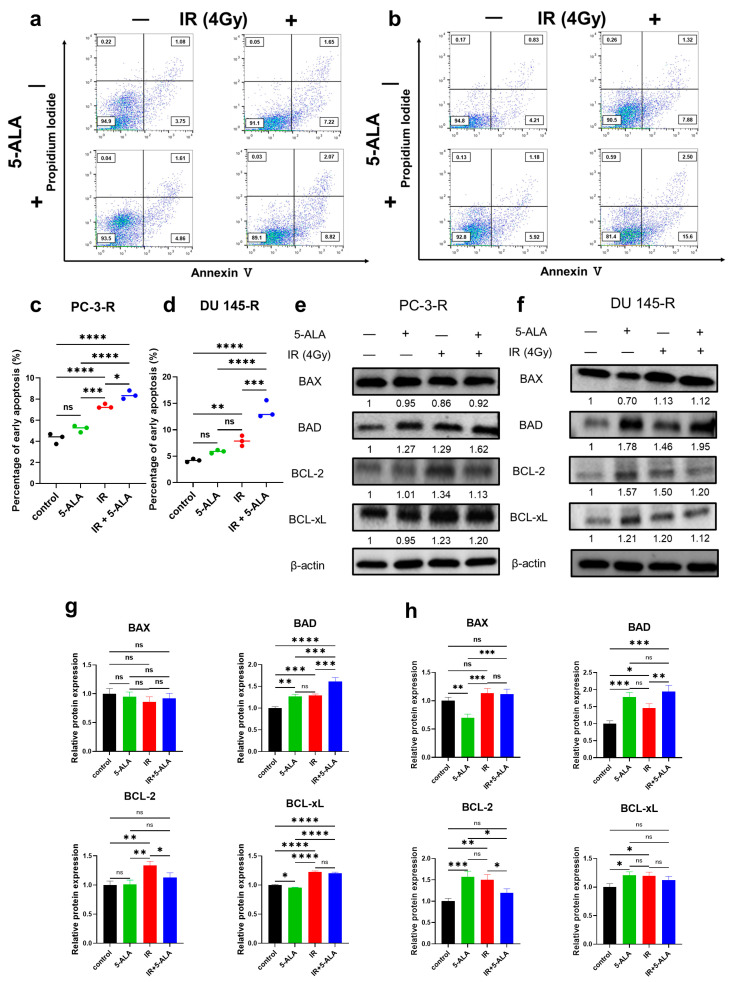
Apoptosis analysis via flow cytometry in radioresistant PCa cells ((**a**,**c**) PC-3-R; (**b**,**d**) DU 145-R). In the radioresistant PCa cells, the expression of BCL-2 family proteins was measured via Western blot analysis ((**e**,**g**) PC-3-R; (**f**,**h**) DU 145-R). 5-ALA: 5-aminolevulinic acid. BAD: BCL-2-associated agonist of cell death. BAX: BCL-2-associated X protein. BCL-2: B-cell/CLL lymphoma 2. BCL-xL: BCL extra-large. IR: irradiation. ns: not significant. PCa: prostate cancer. * *p* < 0.05, ** *p* < 0.01, *** *p* < 0.001, **** *p* < 0.0001. Original western blots are presented in File S1.

Similar to the parental PCa cells, the early apoptosis rates were evaluated in radioresistant PCa cells by dividing them into the following groups: control, 5-ALA-alone, IR-alone, and 5-ALA combined with IR groups. In PC-3-R cells (Figure 2a,c), no significant difference in early apoptosis rate was observed between the 5-ALA-alone group and the control group (control, 4.3 ± 0.4%; 5-ALA alone, 5.2 ± 0.3%; *p* = 0.059). However, the 5-ALA combined with the IR group showed a significant increase in early apoptosis rate compared to the IR-alone group (IR alone, 7.3 ± 0.2%; 5-ALA + IR, 8.4 ± 0.3%; *p* = 0.028). Similarly, in DU 145-R cells (Figure 2b,d), no significant difference was observed between the 5-ALA-alone group and the control group (control, 4.2 ± 0.2%; 5-ALA alone, 5.8 ± 0.3%; *p* = 0.233), but a significant increase in early apoptosis was noted in the 5-ALA combined with the IR group compared to the IR-alone group (IR alone, 7.9 ± 0.8%; 5-ALA + IR, 13.7 ± 1.3%; *p* < 0.001). The combination of 5-ALA and IR induced apoptosis through the radiosensitizing effect of 5-ALA, and this effect was also observed in radioresistant PCa cells.

### 3.5. Effects of 5-ALA on the Expression of Apoptosis-Related Proteins After IR in Parental and Radioresistant PCa Cells

The expression of BCL-2 family proteins, which are mitochondria-mediated apoptosis signaling proteins, was comparatively assessed via Western blotting in the parental and radioresistant PCa cells. The BCL-2 family of proteins consists of pro-apoptotic factors, such as BAX and BAD, and anti-apoptotic factors, such as BCL-2 and BCL-xL. In PC-3 cells (Figure S2e,f), no significant difference was observed in the expression of the pro-apoptotic factors BAX and BAD in the 5-ALA combined with IR group compared to the IR-alone group (BAX, *p* = 0.210; BAD, *p* = 0.202). However, the expression of the anti-apoptotic factors BCL-2 and BCL-xL was significantly reduced in the 5-ALA combined with the IR group compared to the IR-alone group (BCL-2, *p* < 0.001; BCL-xL, *p* = 0.002). In DU 145 cells (Figure S2g,h), no significant difference was observed in the expression of BAX and BCL-2 in the 5-ALA combined with IR group compared to the IR-alone group (BAX, *p* = 0.405; BCL-2, *p* = 0.145). However, significant upregulation of BAD expression and significant downregulation of BCL-xL were observed in the 5-ALA combined with the IR group (BAD, *p* < 0.001; BCL-xL, *p* = 0.002).

In PC-3-R cells (Figure 2e,g), compared to the group treated with IR alone, the group treated with a combination of 5-ALA and IR exhibited significant upregulation of BAD and significant downregulation of BCL-2 (BAD, *p* < 0.004; BCL-2, *p* = 0.035), but no significant difference was observed in BAX and BCL-xL expression between the two groups (BAX, *p* = 0.839; BCL-xL, *p* = 0.511). In the DU 145-R cells (Figure 2f,h), compared to the group treated with IR alone, the group treated with a combination of 5-ALA and IR exhibited significant upregulation of BAD and significant downregulation of BCL-2 (BAD, *p* = 0.010; BCL-2, *p* = 0.013), but no significant difference was observed in BAX and BCL-xL expression between the two groups (BAX, *p* = 0.994; BCL-xL, *p* = 0.547). These findings suggest that the radiosensitizing effect of 5-ALA promotes mitochondria-mediated apoptosis not only in parental PCa cells but also in radioresistant PCa cells.

### 3.6. Difference in the Radiosensitizing Effect of 5-ALA Between Radioresistant PCa Cells and Parental PCa Cells

A comparison of the relative survival curves of the parental and radioresistant PCa cells following treatment with 5-ALA combined with IR revealed that the radioresistant cell lines (PC-3-R (Figure 1c) and DU 145-R (Figure 1d)) exhibited significantly higher survival rates than their parental cell lines (PC-3 and DU 145) (PC-3 vs. PC-3-R, *p* < 0.001; DU 145 vs. DU 145-R, *p* < 0.001). This finding indicates that 5-ALA has a reduced radiosensitizing effect on the radioresistant PCa cells compared to the parental PCa cells.

### 3.7. Effects of 5-ALA on Mitochondrial ROS in Parental and Radioresistant PCa Cells

**Figure 3:**
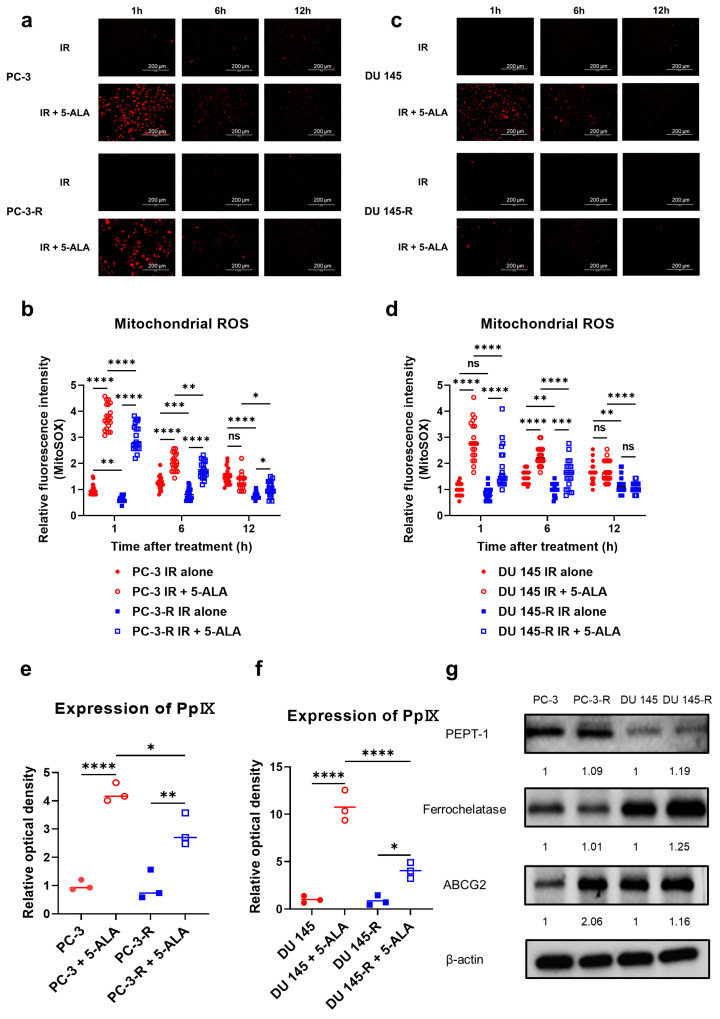
Temporal changes in mitochondrial ROS were compared using a fluorescence microscope in parental and radioresistant PCa cells ((**a**,**b**) PC-3 and PC-3-R; (**c**,**d**) DU 145 and DU 145-R). Intracellular PpIX expression in the parental and radioresistant PCa cells was measured using a microplate spectrophotometer ((**e**) PC-3 and PC-3-R; (**f**) DU 145 and DU 145-R). (**g**) The expression of proteins related to the heme synthesis pathway was measured via Western blot analysis in the parental and radioresistant PCa cells. 5-ALA: 5-aminolevulinic acid. ABCG2: ATP-binding cassette transporter subfamily G2. IR: irradiation. ns: not significant. PCa: prostate cancer. PEPT-1: proton-coupled peptide transporter 1. PpIX: protoporphyrin IX. ROS: reactive oxygen species. * *p* < 0.05, ** *p* < 0.01, *** *p* < 0.001, **** *p* < 0.0001. Original western blots are presented in File S1.

In both PC-3-R (Figure 3a,b) and DU 145-R cells (Figure 3c,d), a significant increase in mitochondrial ROS generation was observed immediately after IR, up to 12 h post-IR, in the combined IR and 5-ALA group compared with the IR-alone group (PC-3-R, 1 h: *p* < 0.001, 6 h: *p* < 0.001, 12 h: *p* = 0.035; DU 145-R, 1 h: *p* < 0.001, 6 h: *p* < 0.001, 12 h: *p* = 0.918). However, mitochondrial ROS generation was significantly lower in radioresistant PCa cells that received 5-ALA with IR than in the parental PCa cells that received 5-ALA with IR at all timepoints up to 12 h after IR, compared with IR alone (PC-3-R, 1 h: *p* < 0.001, 6 h: *p* = 0.010, 12 h: *p* = 0.016; DU 145-R; 1 h: *p* < 0.001, 6 h: *p* < 0.001, 12 h: *p* < 0.001). Additionally, in the IR-alone group, radioresistant PCa cells exhibited significantly lower mitochondrial ROS generation than the parental cells (PC-3-R, 1 h: *p* = 0.001, 6 h; *p* < 0.001, 12 h; *p* < 0.001; DU 145-R, 1 h: *p* = 0.650, 6 h: *p* = 0.003, 12 h: *p* = 0.004). Therefore, while an increase in mitochondrial ROS was observed in the radioresistant PCa cells following 5-ALA treatment combined with IR, similar to the parental cell lines, the production of mitochondrial ROS was significantly lower in the radioresistant PCa cells compared to the parental PCa cells.

### 3.8. Intracellular PpIX Accumulation After Administration of 5-ALA in Parental and Radioresistant PCa Cells

After the administration of 5-ALA in PC-3-R (Figure 3e) and DU 145-R (Figure 3f) cells, a significant increase in PpIX accumulation was observed compared with the control without 5-ALA (PC-3-R, *p* = 0.002; DU 145-R, *p* = 0.012). However, PpIX accumulation after 5-ALA administration was significantly lower in radioresistant PCa cells than in the parental cells (PC-3-R, *p* = 0.015; DU 145-R, *p* < 0.001).

### 3.9. Expression of Proteins Metabolizing 5-ALA in Parental and Radioresistant PCa Cells

The expression of proteins that metabolize 5-ALA was evaluated via Western blotting (Figure 3g). No significant difference was observed in the expression of PEPT-1—a peptide transporter responsible for the cytoplasmic uptake of 5-ALA—between PC-3 and PC-3-R cells (*p* = 0.658), or between DU 145 and DU 145-R cells (*p* = 0.478). The expression of ferrochelatase, which metabolizes mitochondrial PpIX into heme, was significantly higher in DU 145-R cells compared to DU 145 cells (*p* = 0.023), but no significant difference was observed between PC-3 and PC-3-R cells (*p* = 0.940). However, the expression of ABCG2, which exports PpIX outside the mitochondria and the cell wall, was significantly upregulated in the radioresistant PCa cells compared to the parental PCa cells in both PC-3-R (*p* < 0.001) and DU 145-R (*p* = 0.020) cells. These findings suggest that ABCG2 upregulation in radioresistant PCa cells promotes the export of PpIX, leading to reduced mitochondrial ROS production, thereby diminishing the radiosensitizing effect of 5-ALA on radioresistant PCa cells.

### 3.10. ABCG2 Knockdown Using siRNA in Radioresistant PCa Cells

**Figure 4:**
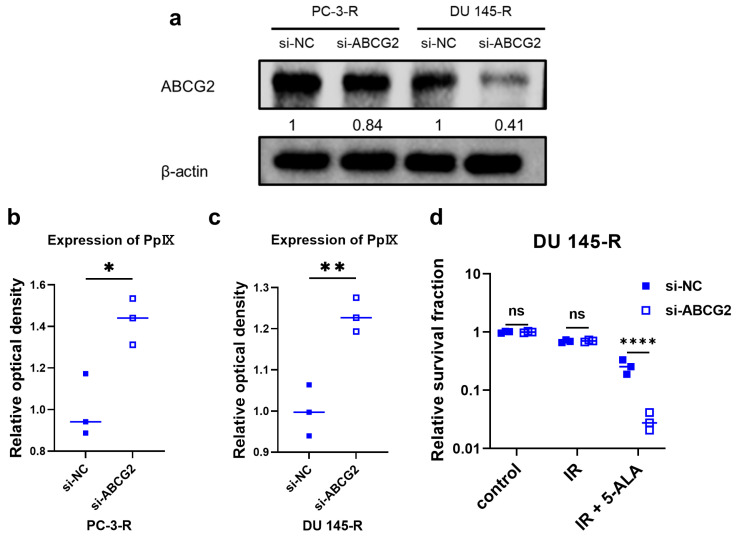
(**a**) ABCG2 expression in radioresistant PCa cells treated with si-NC or si-ABCG2 was measured via Western blot analysis. Intracellular PpIX expression in the radioresistant PCa cells treated with si-NC or si-ABCG2 was measured using a microplate spectrophotometer ((**b**) PC-3-R; (**c**) DU 145-R). (**d**) Relative survival fractions of DU 145-R cells treated with si-NC or si-ABCG2 were compared among each group. 5-ALA: 5-aminolevulinic acid. ABCG2: ATP-binding cassette transporter subfamily G2. IR: irradiation. NC: negative control. ns: not significant. PCa: prostate cancer. PpIX: protoporphyrin IX. * *p* < 0.05, ** *p* < 0.01, **** *p* < 0.0001. Original western blots are presented in File S1.

PC-3-R and DU 145-R cells were treated with either si-NC or si-ABCG2 to knock down ABCG2 expression. Western blot analysis revealed significant downregulation of ABCG2 in both PC-3-R and DU 145-R cells (PC-3-R, *p* = 0.028; DU 145-R, *p* < 0.001) (Figure 4a). PC-3-R (Figure 4b) and DU 145-R (Figure 4c) cells treated with si-ABCG2 showed significantly increased PpIX accumulation after 5-ALA administration compared to the si-NC-treated groups (PC-3-R, *p* = 0.017; DU 145-R, *p* = 0.006). In DU 145-R cells (Figure 4d), si-NC- and si-ABCG2-treated cells were assigned to the control, IR-alone, and 5-ALA combined with IR groups. The relative survival rates were compared using a colony formation assay. A representative image depicting the clonogenic assay results is shown in Figure S1b. No significant differences were observed between the two groups that received IR alone (*p* = 0.993), but the si-ABCG2 group that received 5-ALA treatment combined with IR showed a significantly decreased relative survival rate (*p* < 0.001). However, si-ABCG2-treated PC-3-R cells, including the control group, did not exhibit cell proliferation. Therefore, the colony formation assay could not be performed in PC-3-R cells.

### 3.11. Establishment of Radioresistant PCa Cells from Mice and Radiosensitizing Effects of 5-ALA in Syngeneic Mouse Models

**Figure 5:**
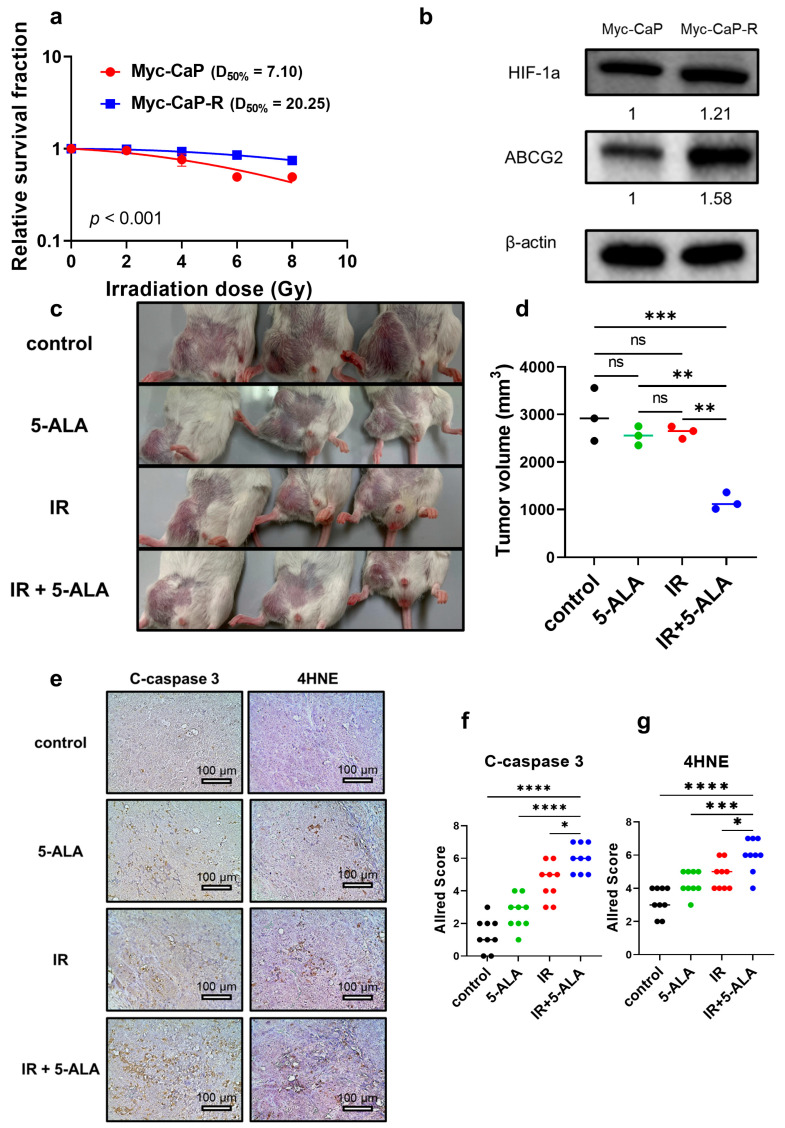
(**a**) Survival curves of the relative survival fractions of Myc-CaP and Myc-CaP-R after IR (2–8 Gy single dose). (**b**) HIF-1a and ABCG2 expression was measured via Western blot analysis in Myc-CaP and Myc-CaP-R cells. (**c**) Photographs of the tumor region inoculated with Myc-CaP-R cells 7 days after treatment in animal experiments, along with (**d**) the actual tumor volumes at the time of euthanasia. (**e**–**g**) C-caspase 3 and 4-HNE expression in the four groups (normal control, 5-ALA alone, IR alone, and a combination of 5-ALA and IR) was evaluated using immunohistochemistry. 4-HNE: 4-hydroxynonenal. 5-ALA: 5-aminolevulinic acid. ABCG2: ATP-binding cassette transporter subfamily G2. C-caspase 3: cleaved caspase 3. D_50%_: radiation dose required to achieve a 50% survival rate. HIF-1a: hypoxia-inducible factor 1a. IR: irradiation. ns: not significant. PCa: prostate cancer. * *p* < 0.05, ** *p* < 0.01, *** *p* < 0.001, **** *p* < 0.0001. Original western blots are presented in File S1.

A mouse-derived radioresistant PCa cell line (Myc-CaP-R) was established from Myc-CaP cells using methods similar to those used for the other PCa cell lines. Compared with Myc-CaP mice, Myc-CaP-R mice exhibited significantly reduced radiosensitivity (*p* < 0.001; Myc-CaP, A value = 0.034, B value = 0.009, D_50%_ = 7.10 Gy; Myc-CaP-R, A value = 0.001, B value = 0.004, D_50%_ = 20.25 Gy; Figure 5a and Figure S1c). Furthermore, similar to the PC-3 and DU 145 cells, the expression of HIF-1a and ABCG2 was evaluated by Western blot analysis. Compared to their expression in Myc-CaP mice, HIF-1a and ABCG2 were significantly upregulated in Myc-CaP-R mice (HIF-1a, *p* = 0.003; ABCG2, *p* = 0.004; Figure 5b).

Following the methods described in the section on the syngeneic mouse model with radioresistant PCa, Myc-CaP-R was inoculated into the mice, and tumor volumes were evaluated 7 days after the completion of treatment in the four groups (control, 5-ALA alone, IR alone, and 5-ALA + IR; Figure 5c,d). No mice died during the course of the animal experiment, and all of the mice were euthanized according to the protocol. The tumor growth curve during the experiment is shown in Figure S1d. Although no significant differences were observed between the control and 5-ALA-alone (*p* = 0.601) or IR-alone groups (*p* = 0.773), the combined 5-ALA and IR treatment group showed a significant reduction in tumor volume compared with the control (*p* = 0.001) and IR-alone groups (*p* = 0.003). Subsequently, the expression of the apoptosis marker C-caspase 3 and the ROS marker 4-HNE in the tumors excised from all groups was compared via immunohistochemistry (Figure 5e–g). The 5-ALA+IR group exhibited significantly upregulated C-caspase 3 expression compared to the control (*p* < 0.001), 5-ALA-alone (*p* < 0.001), and IR-alone groups (*p* = 0.014). Regarding 4-HNE expression, the 5-ALA+IR group showed significantly elevated expression compared to the control (*p* < 0.001), 5-ALA-alone (*p* = 0.001), and IR-alone groups (*p* = 0.014).

### 3.12. HIF-1a and ABCG2 Expression in Human PCa Specimens with Recurrence After Primary RT

**Figure 6:**
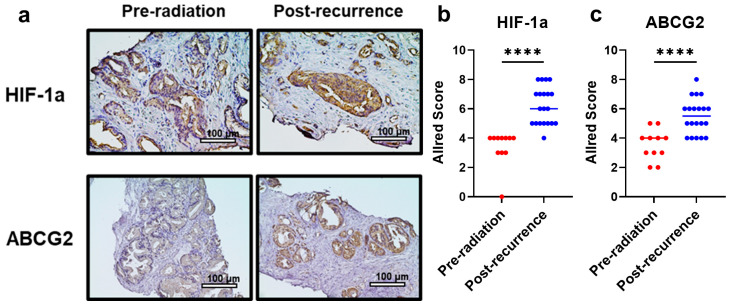
Immunohistochemical evaluation of (**a**,**b**) HIF-1a and (**a**,**c**) ABCG2 expression in human PCa specimens (pre-radiation and post-recurrence) was conducted. ABCG2: ATP-binding cassette transporter subfamily G2. HIF-1a: hypoxia-inducible factor 1a. PCa: prostate cancer. **** *p* < 0.0001.

HIF-1a and ABCG2 expression in human PCa specimens before primary RT and at the time of recurrence after RT was evaluated through immunohistochemistry (Figure 6a). The analysis revealed significantly upregulated HIF-1a expression following recurrence, compared with that before primary RT (*p* < 0.001; Figure 6b). Similarly, ABCG2 was significantly upregulated following recurrence, compared with that before primary RT (*p* < 0.001; Figure 6c).

## 4. Discussion

We previously reported the radiosensitizing effects of 5-ALA on PCa cells [24,25]. Nevertheless, this study is the first to demonstrate that 5-ALA also increases radiosensitivity in radioresistant PCa cells, which was established by consecutive irradiation. The established radioresistant PCa cells exhibited reduced radiosensitivity compared to the parental PCa cells. One possible mechanism underlying radioresistance is the reduction in mitochondrial ROS generation following IR. Additionally, upregulation of HIF-1a and HIF-2a was evident in radioresistant PCa cells and recurrent tumors following RT, suggesting hypoxic conditions within the tumor microenvironment. Solid tumors create hypoxic niches due to their stromal composition, tumor growth, and aberrant vascularization [14]. In PCa cells, hypoxia within the tumor microenvironment promotes angiogenesis and increases tumor cells’ migration and invasion, contributing to cancer progression [39,40]. Additionally, hypoxia-induced suppression of ROS production has been linked to resistance not only to radiation but also to chemotherapy and apoptosis [41,42]. This interplay underscores the multifaceted nature of radioresistance mechanisms in PCa [43]. Moreover, other studies have shown that radiosensitivity is approximately three times higher in well-oxygenated environments compared to hypoxic conditions [44]. Therefore, the reduced radiosensitivity in radioresistant PCa cells stems from hypoxia in the tumor microenvironment. HIF-1a stands out as a primary hypoxic marker, triggering the expression of crucial factors such as vascular endothelial growth factor, which is pivotal in angiogenesis, and glucose transporter 1, indicating anaerobic metabolism [14,45]. Consequently, HIF-1a expression is a factor that is considered to reduce radiosensitivity and is involved in the mechanisms underlying the acquisition of radioresistance [15]. Other reports have indicated that HIF-1a expression in PCa tissues at the time of diagnosis was not associated with prognosis [46]. In this study, significantly increased HIF-1a expression was observed at the time of local recurrence after RT. Therefore, HIF-1a represents a promising therapeutic target in PCa, not only for increasing radiosensitivity but also for developing novel strategies to prevent the acquisition of radioresistance [41].

In established radioresistant PCa cells, the administration of 5-ALA increased their radiosensitivity, indicating the radiosensitizing effect of 5-ALA in radioresistant PCa cells. We have previously reported the radiosensitizing effect of 5-ALA in parental PCa cells under normoxic and hypoxic conditions [24,25]. The mechanism underlying the radiosensitizing effect of 5-ALA involves the increased generation of mitochondrial ROS following IR, leading to PpIX accumulation within the mitochondria of cancer cells, which promotes apoptosis [24]. In this study, the combination of 5-ALA and RT significantly increased PpIX accumulation within the mitochondria and mitochondrial ROS generation in radioresistant PCa cells compared with RT alone. This accumulation resulted in the upregulation of the mitochondria-mediated pro-apoptotic factors BAX and BAD, leading to apoptosis. Similar results were observed in vivo, suggesting the potential of 5-ALA to increase radiosensitivity in radioresistant PCa cells. Additionally, it has been reported that 5-ALA activates mitochondrial function in normal cells and exerts anti-inflammatory effects, thereby suppressing cisplatin-induced kidney injury [47]. In our previous study, mucosal-protective effects of 5-ALA were reported in the bladders and recta of mice within the radiation-exposed area in the group administered 5-ALA, without any significant adverse effects [25]. Thus, 5-ALA administration may be a safe, effective, and novel therapeutic strategy for treating radioresistant PCa. However, clinical trials have not yet been conducted, and the efficacy and safety of 5-ALA in salvage RT for recurrent cases after primary RT remains unknown.

The radiosensitizing effect of 5-ALA was significantly lower in the radioresistant PCa cells than in the parental PCa cells. This decline was attributed to reduced mitochondrial ROS production due to decreased PpIX accumulation. The decline in PpIX accumulation was linked to heightened expression of the drug transporter ABCG2. ABCG2, also known as a breast cancer resistance protein, resides on the mitochondrial and cellular membranes [48]. ABCG2 can decrease the effectiveness of PDT with 5-ALA via extracellular discharge of PpIX [49,50]. Furthermore, ABCG2 functions as a drug transporter and discharges cytotoxic substances, such as anticancer agents and ROS, and ABCG2 upregulation has been associated with the acquisition of resistance to RT and chemotherapy, as well as with stemness [48,51]. Consequently, numerous novel therapeutic strategies targeting ABCG2 have been reported in recent years [52,53]. Indeed, ABCG2 inhibitors have been shown to increase radiosensitivity [54]. However, their clinical use is hindered by severe neurological side effects observed in vivo, since ABCG2 is also responsible for eliminating cytotoxic substances in normal cells [55]. In this study, ABCG2 knockdown using siRNA resulted in increased PpIX accumulation, thereby enhancing the radiosensitizing effect of 5-ALA. However, in PC-3-R cells, ABCG2 knockdown led to an absence of cell proliferation, indicating that targeting ABCG2 in future therapies will require careful consideration of potential effects on normal cells.

This study has several limitations. Firstly, although the radiosensitizing effect of 5-ALA was evident in vivo and in mice, its clinical translation remains uncertain. Secondly, in this study, radioresistant PCa cells were established through prolonged consecutive IR. However, the possibility that long-term cell culture contributed to the acquisition of radioresistance cannot be entirely excluded. Previous studies have reported that prolonged cell culture can induce epigenetic changes, including the acquisition of radiation resistance [56]. Nevertheless, it has also been reported that radioresistant cancer cells lose their resistance within approximately six months to one year after discontinuing continuous IR [57]. This suggests that continuous exposure to radiation plays a significant role in the acquisition of radioresistance. While an appropriate control group for this study might ideally consist of PCa cells cultured for the same duration, the radioresistant PCa cells established in this study exhibited low radiosensitivity compared to the control group. Therefore, the findings of this study are not fundamentally undermined, and it was considered appropriate to evaluate the radiosensitizing effect of 5-ALA on radioresistant PCa cells in this study. Furthermore, in this study, cell treatment after 5-ALA administration was performed under light-shielded conditions to the greatest extent possible. However, PpIX can induce PDT and generate mitochondrial ROS even under minimal visible-light exposure [30]. Therefore, it cannot be completely ruled out that the results obtained in this study were influenced by PDT effects, making the quality of light shielding a potential limitation of this study. Nevertheless, as all groups were treated equally in all experiments, the combination of 5-ALA and IR demonstrated enhanced antitumor effects compared to either 5-ALA alone or IR alone, confirming the radiosensitizing effect of 5-ALA. Then, in this study, mitochondrial ROS was evaluated using MitoSOX Red, which specifically measures mitochondrial superoxide rather than hydroxyl radicals. Additionally, according to the product protocol, the excitation wavelength is 396 nm and the emission wavelength is 580 nm. Therefore, observation using the RFP light cube may potentially detect non-specific signals, which we consider to be a potential limitation. Finally, the number of mice used in the animal experiments and the number of human specimens were limited, which likely resulted in insufficient statistical power. Furthermore, since the human specimens were obtained from biopsy samples, the small tumor volume restricted thorough investigation.

## 5. Conclusions

In conclusion, radioresistant PCa cells exhibited a hypoxic signaling environment characterized by the upregulation of HIF-1a (PC-3-R, *p* < 0.001; DU 145-R, *p* = 0.030; Myc-CaP-R, *p* = 0.003) and HIF-2a (PC-3-R, *p* < 0.001; DU 145-R, *p* = 0.034) compared to their parental PCa cells, resulting in reduced radiosensitivity (PC-3, *p* < 0.001; DU 145-R, *p* < 0.001; Myc-CaP-R, *p* < 0.001). However, the combination of 5-ALA and RT significantly increased radiosensitivity even in radioresistant PCa cells (PC-3-R, *p* < 0.001; DU 145-R, *p* < 0.001). Nevertheless, in the radioresistant PCa cells, the radiosensitizing effect of 5-ALA was limited compared to the parental PCa cells (PC-3-R, *p* < 0.001; DU 145-R, *p* < 0.001), which may be attributable to the upregulation of the drug transporter ABCG2 in radioresistant PCa cells (PC-3-R, *p* < 0.001; DU 145-R, *p* = 0.020; Myc-CaP-R, *p* = 0.004). Although challenges remain in overcoming radioresistance, combining 5-ALA with focal therapies, such as salvage low-dose-rate brachytherapy for locally recurrent PCa after primary radical RT, shows promise due to its low toxicity. Thus, further clinical studies are warranted.

## Data Availability

The data generated in the present study may be requested from the corresponding author.

## Supplementary Materials

The following supporting information can be downloaded at https://www.mdpi.com/article/10.3390/cancers17081286/s1, **Figure S1**: (**a**) Representative images of the clonogenic assay shown in Figure 1c,d. (**b**) Representative images of the clonogenic assay shown in Figure 4d. (**c**) Representative images of the clonogenic assay shown in Figure 5a. (**d**) Alteration of tumor volume in animal experiments. 5-ALA: 5-aminolevulinic acid. ABCG2: ATP-binding cassette transporter subfamily G2. IR: irradiation. NC: negative control. PCa: prostate cancer. **Figure S2**: Apoptosis analysis via flow cytometry in the parental PCa cells ((**a,b**) PC-3; (**c,d**) DU 145). In the parental PCa cells, the expression of BCL-2 family proteins was measured via Western blot analysis ((**e,f**) PC-3; (**g,h**) DU 145). 5-ALA: 5-aminolevulinic acid. BAD: BCL-2-associated agonist of cell death. BAX: BCL-2-associated X protein. BCL-2: B-cell/CLL lymphoma 2. BCL-xL: BCL extra-large. IR: irradiation. ns: not significant. PCa: prostate cancer. ** *p* < 0.01, *** *p* < 0.001. File S1: Original western blots.

## Abbreviations

The following abbreviations are used in this manuscript:

4-HNE	4-Hydroxynonenal
5-ALA	5-Aminolevulinic acid
ABCG2	ATP-binding cassette transporter subfamily G2
BAD	BCL-2-associated agonist of cell death
BAX	BCL-2-associated X protein
BCL-xL	BCL extra-large
BCL-2	B-cell/CLL lymphoma 2
C-caspase 3	Cleaved caspase 3
D_50%_	Radiation dose required to achieve a 50% survival rate
HIF-1a	Hypoxia-inducible-factor 1a
IC_50_	Half-maximal inhibitory concentration
IR	Irradiation
PCa	Prostate cancer
PDT	Photodynamic therapy
PpIX	Protoporphyrin IX
ROS	Reactive oxygen species
RT	Radiation therapy

## References

[1] Sung H., Ferlay J., Siegel R.L., Laversanne M., Soerjomataram I., Jemal A., Bray F. (2021). Global Cancer Statistics 2020: GLOBOCAN Estimates of Incidence and Mortality Worldwide for 36 Cancers in 185 Countries. CA Cancer J Clin..

[2] Gentile F., La Civita E., Della Ventura B., Ferro M., Cennamo M., Bruzzese D., Crocetto F., Velotta R., Terracciano D. (2022). A Combinatorial Neural Network Analysis Reveals a Synergistic Behaviour of Multiparametric Magnetic Resonance and Prostate Health Index in the Identification of Clinically Significant Prostate Cancer. Clin. Genitourin. Cancer.

[3] Di Lorenzo G., Buonerba L., Ingenito C., Crocetto F., Buonerba C., Libroia A., Sciarra A., Ragone G., Sanseverino R., Iaccarino S. (2020). Clinical Characteristics of Metastatic Prostate Cancer Patients Infected with COVID-19 in South Italy. Oncology.

[4] Sekhoacha M., Riet K., Motloung P., Gumenku L., Adegoke A., Mashele S. (2022). Prostate Cancer Review: Genetics, Diagnosis, Treatment Options, and Alternative Approaches. Molecules.

[5] Grimm P., Billiet I., Bostwick D., Dicker A.P., Frank S., Immerzeel J., Keyes M., Kupelian P., Lee W.R., Machtens S. (2012). Comparative analysis of prostate-specific antigen free survival outcomes for patients with low, intermediate and high risk prostate cancer treatment by radical therapy. Results from the Prostate Cancer Results Study Group. BJU Int..

[6] Merrick G.S., Butler W.M., Galbreath R.W., Lief J., Bittner N., Wallner K.E., Adamovich E. (2011). Prostate cancer death is unlikely in high-risk patients following quality permanent interstitial brachytherapy. BJU Int..

[7] Kuban D.A., Levy L.B., Cheung M.R., Lee A.K., Choi S., Frank S., Pollack A. (2011). Long-term failure patterns and survival in a randomized dose-escalation trial for prostate cancer. Who dies of disease?. Int. J. Radiat. Oncol. Biol. Phys..

[8] Deek M., Lilleby W., Vaage V., Hole K.H., DeWeese T., Stensvold A., Tran P., Seierstad T. (2020). Impact of radiation dose on recurrence in high-risk prostate cancer patients. Prostate.

[9] Miyake M., Tanaka N., Asakawa I., Owari T., Hori S., Morizawa Y., Nakai Y., Inoue T., Anai S., Torimoto K. (2019). The impact of the definition of biochemical recurrence following salvage radiotherapy on outcomes and prognostication in patients with recurrent prostate cancer after radical prostatectomy: A comparative study of three definitions. Prostate Int..

[10] Zumsteg Z.S., Spratt D.E., Romesser P.B., Pei X., Zhang Z., Kollmeier M., McBride S., Yamada Y., Zelefsky M.J. (2015). Anatomical Patterns of Recurrence Following Biochemical Relapse in the Dose Escalation Era of External Beam Radiotherapy for Prostate Cancer. J. Urol..

[11] Henderson R.H., Bryant C., Nichols R.C., Mendenhall W.M., Mendenhall N.P. (2023). Local salvage of radiorecurrent prostate cancer. Prostate.

[12] Valle L.F., Lehrer E.J., Markovic D., Elashoff D., Levin-Epstein R., Karnes R.J., Reiter R.E., Rettig M., Calais J., Nickols N.G. (2021). A Systematic Review and Meta-analysis of Local Salvage Therapies After Radiotherapy for Prostate Cancer (MASTER). Eur. Urol..

[13] Macedo-Silva C., Benedetti R., Ciardiello F., Cappabianca S., Jeronimo C., Altucci L. (2021). Epigenetic mechanisms underlying prostate cancer radioresistance. Clin. Epigenet..

[14] Bouleftour W., Rowinski E., Louati S., Sotton S., Wozny A.S., Moreno-Acosta P., Mery B., Rodriguez-Lafrasse C., Magne N. (2021). A Review of the Role of Hypoxia in Radioresistance in Cancer Therapy. Med. Sci. Monit..

[15] Lee D.E., Alhallak K., Jenkins S.V., Vargas I., Greene N.P., Quinn K.P., Griffin R.J., Dings R.P.M., Rajaram N. (2018). A Radiosensitizing Inhibitor of HIF-1 alters the Optical Redox State of Human Lung Cancer Cells In Vitro. Sci. Rep..

[16] Li F., Zhou K., Gao L., Zhang B., Li W., Yan W., Song X., Yu H., Wang S., Yu N. (2016). Radiation induces the generation of cancer stem cells: A novel mechanism for cancer radioresistance. Oncol. Lett..

[17] Keam S.P., Caramia F., Gamell C., Paul P.J., Arnau G.M., Neeson P.J., Williams S.G., Haupt Y. (2018). The Transcriptional Landscape of Radiation-Treated Human Prostate Cancer: Analysis of a Prospective Tissue Cohort. Int. J. Radiat. Oncol. Biol. Phys..

[18] Gravina G.L., Marampon F., Sherris D., Vittorini F., Di Cesare E., Tombolini V., Lenzi A., Jannini E.A., Festuccia C. (2014). Torc1/Torc2 inhibitor, Palomid 529, enhances radiation response modulating CRM1-mediated survivin function and delaying DNA repair in prostate cancer models. Prostate.

[19] Miyake M., Ishii M., Kawashima K., Kodama T., Sugano K., Fujimoto K., Hirao Y. (2009). siRNA-mediated knockdown of the heme synthesis and degradation pathways: Modulation of treatment effect of 5-aminolevulinic acid-based photodynamic therapy in urothelial cancer cell lines. Photochem. Photobiol..

[20] Kennedy J.C., Pottier R.H. (1992). Endogenous protoporphyrin IX, a clinically useful photosensitizer for photodynamic therapy. J. Photochem. Photobiol. B.

[21] Chan K.M., Gleadle J., Vasilev K., MacGregor M. (2020). Probing Hexaminolevulinate Mediated PpIX Fluorescence in Cancer Cell Suspensions in the Presence of Chemical Adjuvants. Int. J. Mol. Sci..

[22] Suzuki K., Yamamoto J., Toh K., Miyaoka R. (2023). 5-aminiolevulinic acid induces a radiodynamic effect with enhanced delayed reactive oxygen species production under hypoxic conditions in lymphoma cells: An in vitro study. Exp. Ther. Med..

[23] Pepper N.B., Eich H.T., Müther M., Oertel M., Rehn S., Spille D.C., Stummer W. (2024). ALA-RDT in GBM: Protocol of the phase I/II dose escalation trial of radiodynamic therapy with 5-Aminolevulinic acid in patients with recurrent glioblastoma. Radiat. Oncol..

[24] Owari T., Tanaka N., Nakai Y., Miyake M., Anai S., Kishi S., Mori S., Fujiwara-Tani R., Hojo Y., Mori T. (2022). 5-Aminolevulinic acid overcomes hypoxia-induced radiation resistance by enhancing mitochondrial reactive oxygen species production in prostate cancer cells. Br. J. Cancer.

[25] Miyake M., Tanaka N., Hori S., Ohnishi S., Takahashi H., Fujii T., Owari T., Ohnishi K., Iida K., Morizawa Y. (2019). Dual benefit of supplementary oral 5-aminolevulinic acid to pelvic radiotherapy in a syngenic prostate cancer model. Prostate.

[26] Ikeda T., Kurokawa H., Ito H., Tsuchiya K., Matsui H. (2024). Enhancement of cytotoxic effects with ALA-PDT on treatment of radioresistant cancer cells. J. Clin. Biochem. Nutr..

[27] Kaighn M.E., Narayan K.S., Ohnuki Y., Lechner J.F., Jones L.W. (1979). Establishment and characterization of a human prostatic carcinoma cell line (PC-3). Investig. Urol..

[28] Stone K.R., Mickey D.D., Wunderli H., Mickey G.H., Paulson D.F. (1978). Isolation of a human prostate carcinoma cell line (DU 145). Int. J. Cancer.

[29] Watson P.A., Ellwood-Yen K., King J.C., Wongvipat J., Lebeau M.M., Sawyers C.L. (2005). Context-dependent hormone-refractory progression revealed through characterization of a novel murine prostate cancer cell line. Cancer Res..

[30] Yang X., Palasuberniam P., Kraus D., Chen B. (2015). Aminolevulinic Acid-Based Tumor Detection and Therapy: Molecular Mechanisms and Strategies for Enhancement. Int. J. Mol. Sci..

[31] Stummer W., Stocker S., Wagner S., Stepp H., Fritsch C., Goetz C., Goetz A.E., Kiefmann R., Reulen H.J. (1998). Intraoperative detection of malignant gliomas by 5-aminolevulinic acid-induced porphyrin fluorescence. Neurosurgery.

[32] McMahon S.J. (2018). The linear quadratic model: Usage, interpretation and challenges. Phys. Med. Biol..

[33] Kuwahara Y., Tomita K., Urushihara Y., Sato T., Kurimasa A., Fukumoto M. (2018). Association between radiation-induced cell death and clinically relevant radioresistance. Histochem. Cell Biol..

[34] Kuwahara Y., Roudkenar M.H., Urushihara Y., Saito Y., Tomita K., Roushandeh A.M., Sato T., Kurimasa A., Fukumoto M. (2017). Clinically relevant radioresistant cell line: A simple model to understand cancer radioresistance. Med. Mol. Morphol..

[35] Kuwahara Y., Li L., Baba T., Nakagawa H., Shimura T., Yamamoto Y., Ohkubo Y., Fukumoto M. (2009). Clinically relevant radioresistant cells efficiently repair DNA double-strand breaks induced by X-rays. Cancer Sci..

[36] Nakai Y., Tatsumi Y., Hori S., Morizawa Y., Iida K., Onishi K., Miyake M., Oda Y., Owari T., Fujii T. (2022). 5-Aminolevurinic acid inhibits the proliferation of bladder cancer cells by activating heme synthesis. Oncol. Rep..

[37] Miyake M., Nakai Y., Anai S., Tatsumi Y., Kuwada M., Onishi S., Chihara Y., Tanaka N., Hirao Y., Fujimoto K. (2014). Diagnostic approach for cancer cells in urine sediments by 5-aminolevulinic acid-based photodynamic detection in bladder cancer. Cancer Sci..

[38] Allred D.C., Harvey J.M., Berardo M., Clark G.M. (1998). Prognostic and predictive factors in breast cancer by immunohistochemical analysis. Mod. Pathol..

[39] Yamamoto D., Sasaki K., Kosaka T., Oya M., Sato T. (2022). Functional analysis of GCNT3 for cell migration and EMT of castration-resistant prostate cancer cells. Glycobiology.

[40] Geng H., Xue C., Mendonca J., Sun X.X., Liu Q., Reardon P.N., Chen Y., Qian K., Hua V., Chen A. (2018). Interplay between hypoxia and androgen controls a metabolic switch conferring resistance to androgen/AR-targeted therapy. Nat. Commun..

[41] Ma S., Zhao Y., Lee W.C., Ong L.T., Lee P.L., Jiang Z., Oguz G., Niu Z., Liu M., Goh J.Y. (2022). Hypoxia induces HIF1α-dependent epigenetic vulnerability in triple negative breast cancer to confer immune effector dysfunction and resistance to anti-PD-1 immunotherapy. Nat. Commun..

[42] Arriagada C., Silva P., Torres V.A. (2019). Role of glycosylation in hypoxia-driven cell migration and invasion. Cell Adhes. Migr..

[43] Carmeliet P., Jain R.K. (2011). Principles and mechanisms of vessel normalization for cancer and other angiogenic diseases. Nat. Rev. Drug Discov..

[44] Rockwell S., Dobrucki I.T., Kim E.Y., Marrison S.T., Vu V.T. (2009). Hypoxia and radiation therapy: Past history, ongoing research, and future promise. Curr. Mol. Med..

[45] De Palma M., Biziato D., Petrova T.V. (2017). Microenvironmental regulation of tumour angiogenesis. Nat. Rev. Cancer.

[46] Wilkins A., Gusterson B., Tovey H., Griffin C., Stuttle C., Daley F., Corbishley C.M., Dearnaley D., Hall E., Somaiah N. (2023). Multi-candidate immunohistochemical markers to assess radiation response and prognosis in prostate cancer: Results from the CHHiP trial of radiotherapy fractionation. EBioMedicine.

[47] Terada Y., Inoue K., Matsumoto T., Ishihara M., Hamada K., Shimamura Y., Ogata K., Inoue K., Taniguchi Y., Horino T. (2013). 5-Aminolevulinic acid protects against cisplatin-induced nephrotoxicity without compromising the anticancer efficiency of cisplatin in rats in vitro and in vivo. PLoS ONE.

[48] Westover D., Li F. (2015). New trends for overcoming ABCG2/BCRP-mediated resistance to cancer therapies. J. Exp. Clin. Cancer Res..

[49] Zhou S., Zong Y., Ney P.A., Nair G., Stewart C.F., Sorrentino B.P. (2005). Increased expression of the Abcg2 transporter during erythroid maturation plays a role in decreasing cellular protoporphyrin IX levels. Blood.

[50] Yamamoto S., Fukuhara H., Seki H., Kawada C., Nakayama T., Karashima T., Ogura S.I., Inoue K. (2021). Predictors of therapeutic efficacy of 5-aminolevulinic acid-based photodynamic therapy in human prostate cancer. Photodiagn. Photodyn. Ther..

[51] Mozner O., Bartos Z., Zambo B., Homolya L., Hegedus T., Sarkadi B. (2019). Cellular Processing of the ABCG2 Transporter-Potential Effects on Gout and Drug Metabolism. Cells.

[52] Samant M.D., Jackson C.M., Felix C.L., Jones A.J., Goodrich D.W., Foster B.A., Huss W.J. (2015). Multi-Drug Resistance ABC Transporter Inhibition Enhances Murine Ventral Prostate Stem/Progenitor Cell Differentiation. Stem Cells Dev..

[53] Ding R., Jin S., Pabon K., Scotto K.W. (2016). A role for ABCG2 beyond drug transport: Regulation of autophagy. Autophagy.

[54] Ingram W.J., Crowther L.M., Little E.B., Freeman R., Harliwong I., Veleva D., Hassall T.E., Remke M., Taylor M.D., Hallahan A.R. (2013). ABC transporter activity linked to radiation resistance and molecular subtype in pediatric medulloblastoma. Exp. Hematol. Oncol..

[55] Gardner E.R., Smith N.F., Figg W.D., Sparreboom A. (2009). Influence of the dual ABCB1 and ABCG2 inhibitor tariquidar on the disposition of oral imatinib in mice. J. Exp. Clin. Cancer Res..

[56] Upadhyaya P., Di Serafino A., Sorino L., Ballerini P., Marchisio M., Pierdomenico L., Stuppia L., Antonucci I. (2019). Genetic and epigenetic modifications induced by chemotherapeutic drugs: Human amniotic fluid stem cells as an in-vitro model. BMC Med. Genom..

[57] Tomita K., Nagasawa T., Kuwahara Y., Torii S., Igarashi K., Roudkenar M.H., Roushandeh A.M., Kurimasa A., Sato T. (2021). MiR-7-5p Is Involved in Ferroptosis Signaling and Radioresistance Thru the Generation of ROS in Radioresistant HeLa and SAS Cell Lines. Int. J. Mol. Sci..

