# Supplementary material for: 5-Aminolevulinic Acid: A Novel Approach to Improving Radioresistance in Prostate Cancer
Source: Cancers (Basel). 2025 Apr 10;17(8):1286. doi: 10.3390/cancers17081286 (PMC12025751; doi:10.3390/cancers17081286)
Supplement: Supplementary file 1 [file cancers-17-01286-s001.zip › cancers-3536058-supplementary.pdf]

Supplementary Materials

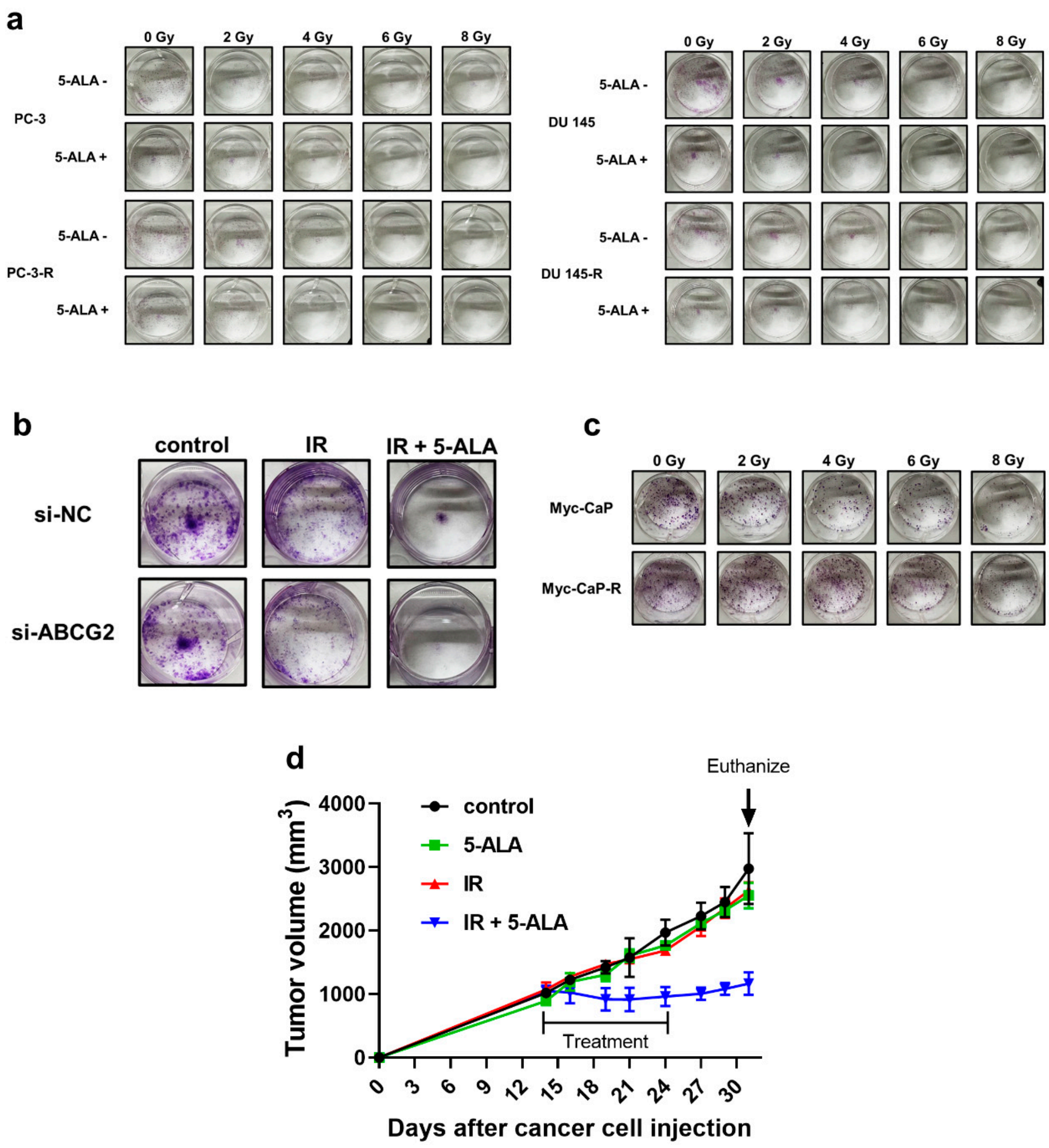

Figure S1: (**a**) Representative images of the clonogenic assay shown in Figure 1c,d. (**b**) Representative images of the clonogenic assay shown in Figure 4d. (**c**) Representative images of the clonogenic assay shown in Figure 5a. (**d**) Alteration of tumor volume in animal experiments. 5-ALA: 5-aminolevulinic acid. ABCG2: ATP-binding cassette transporter subfamily G2. IR: irradiation. NC: negative control. PCa: prostate cancer.

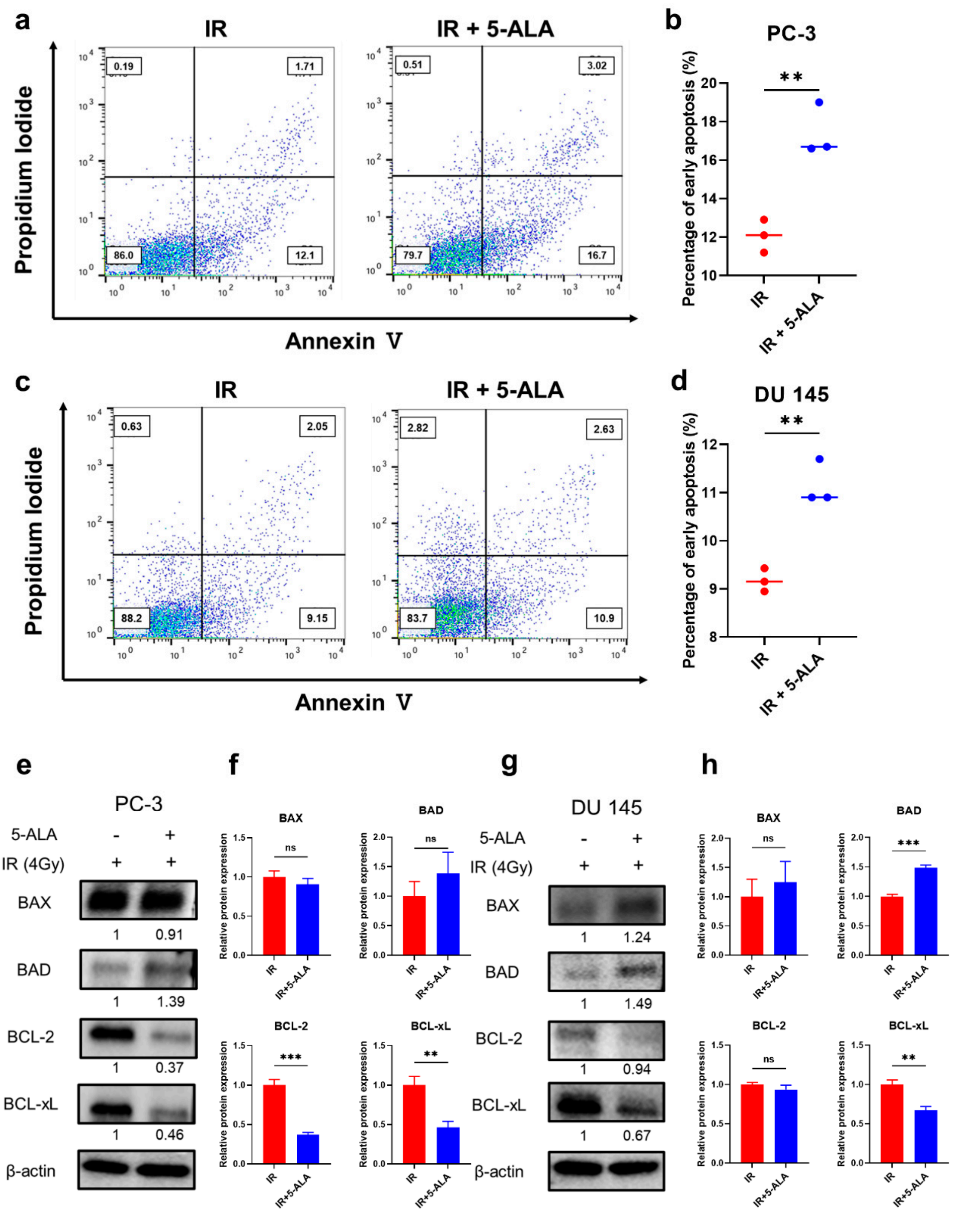

Figure S2: Apoptosis analysis via flow cytometry in the parental PCa cells ((**a, b**) PC-3; (**c, d**) DU 145). In the parental PCa cells, the expression of BCL-2 family proteins was measured via Western blot analysis ((**e, f**) PC-3; (**g, h**) DU 145). 5-ALA: 5-aminolevulinic acid. BAD: BCL-2-associated agonist of cell death. BAX: BCL-2-associated X protein. BCL-2: B-cell/CLL lymphoma 2. BCL-xL: BCL extra-large. IR: irradiation. ns: not significant. PCa: prostate cancer. $^{**}p < 0.01$, $^{***}p < 0.001$.
