# Supplementary figures and images for: 5-Aminolevulinic Acid: A Novel Approach to Improving Radioresistance in Prostate Cancer
Source: Cancers (Basel). 2025 Apr 10;17(8):1286. doi: 10.3390/cancers17081286 (PMC12025751; doi:10.3390/cancers17081286)

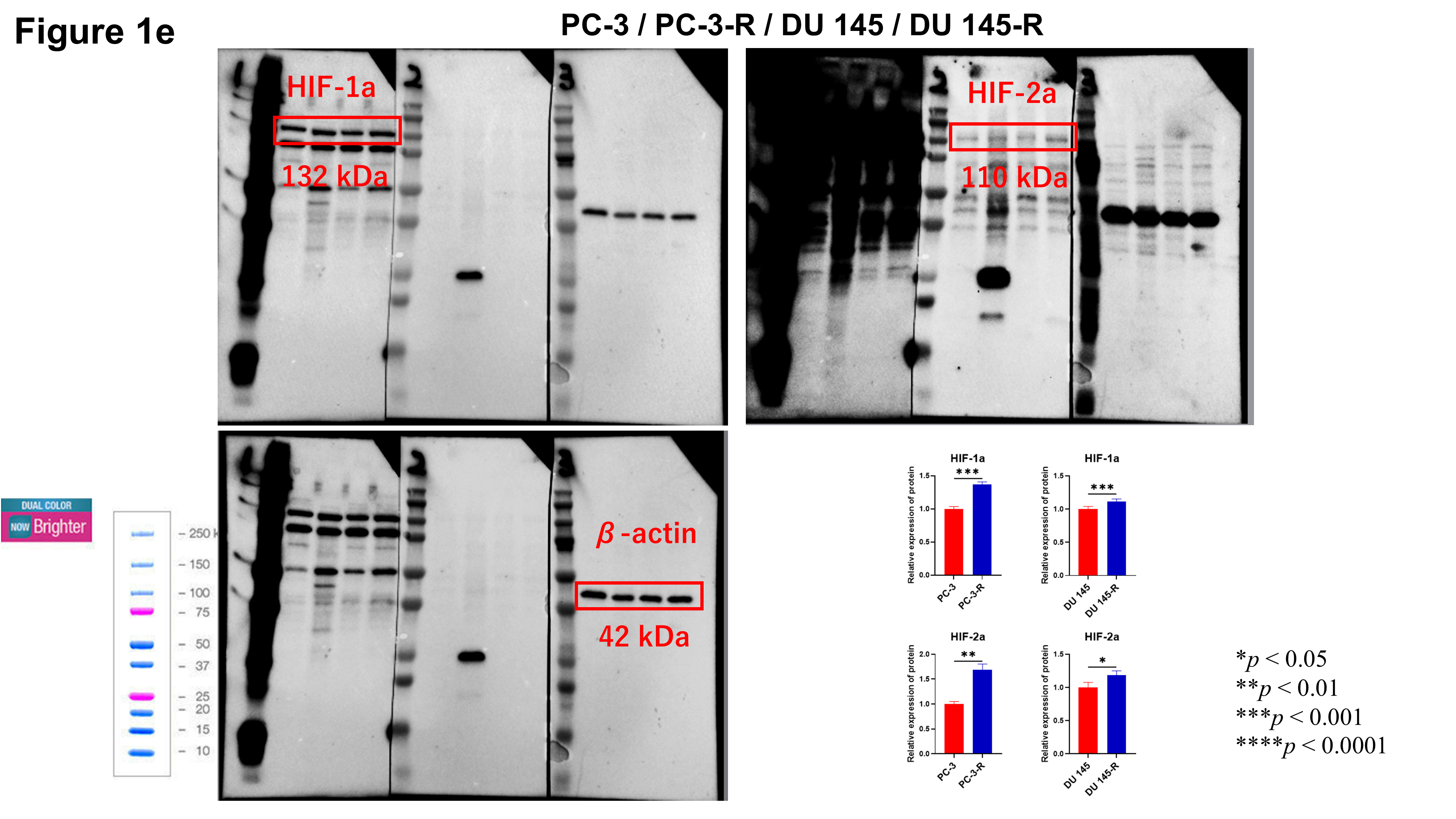

Supplement: Supplementary file 1 [file cancers-17-01286-s001.zip › cancers-3536058-File S1/Figure 1e.TIF]

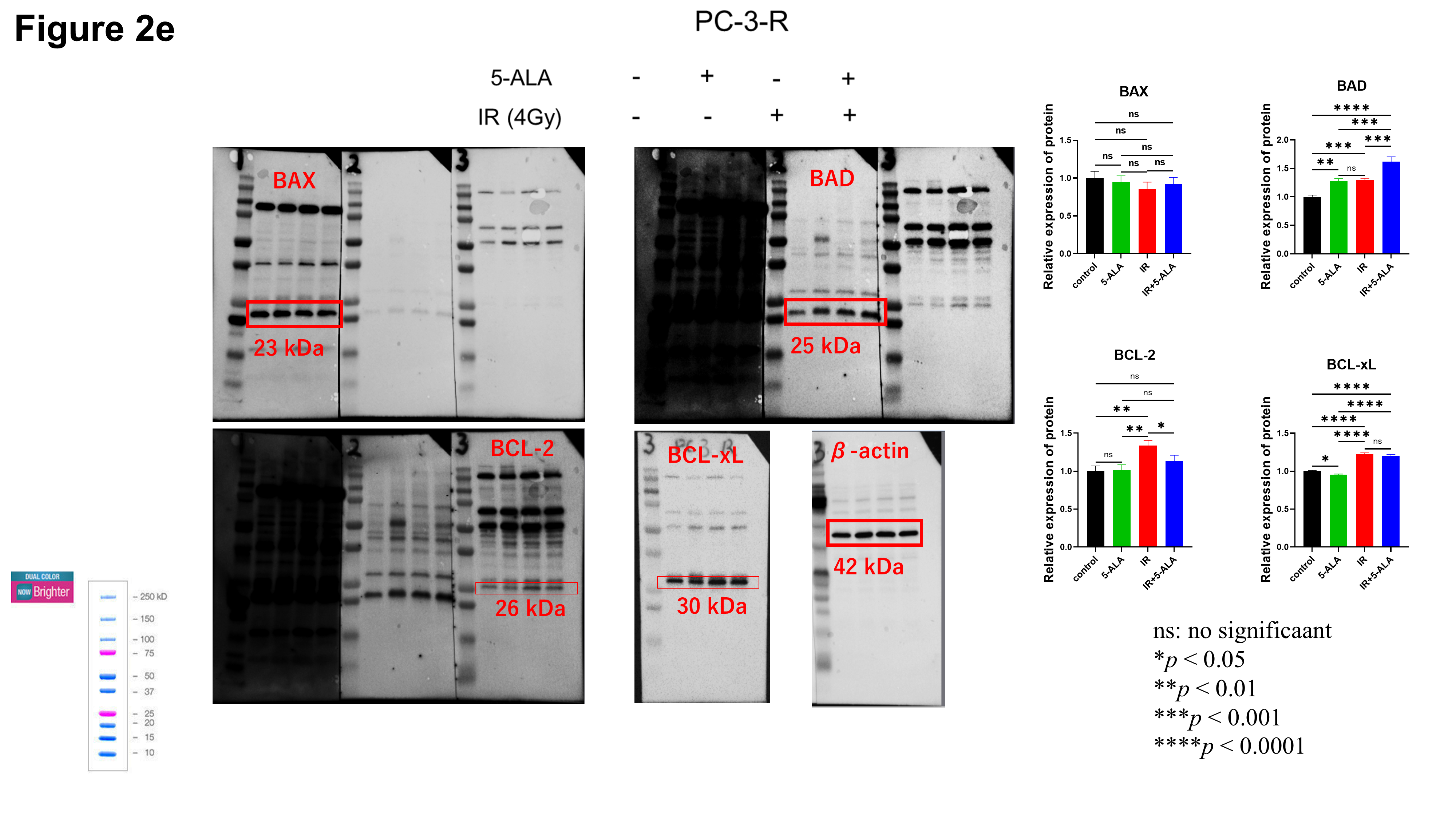

Supplement: Supplementary file 1 [file cancers-17-01286-s001.zip › cancers-3536058-File S1/Figure 2e.TIF]

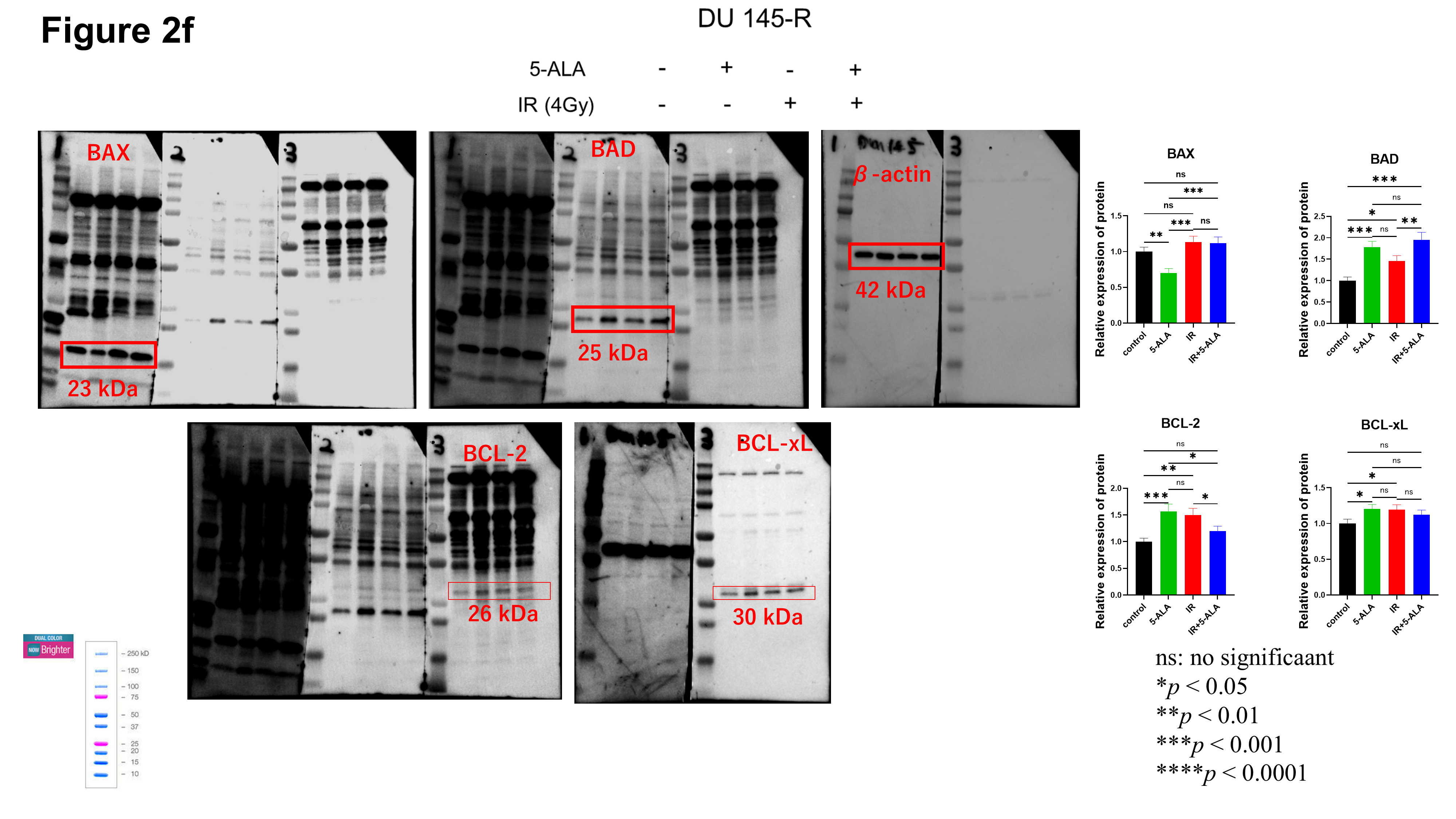

Supplement: Supplementary file 1 [file cancers-17-01286-s001.zip › cancers-3536058-File S1/Figure 2f.TIF]

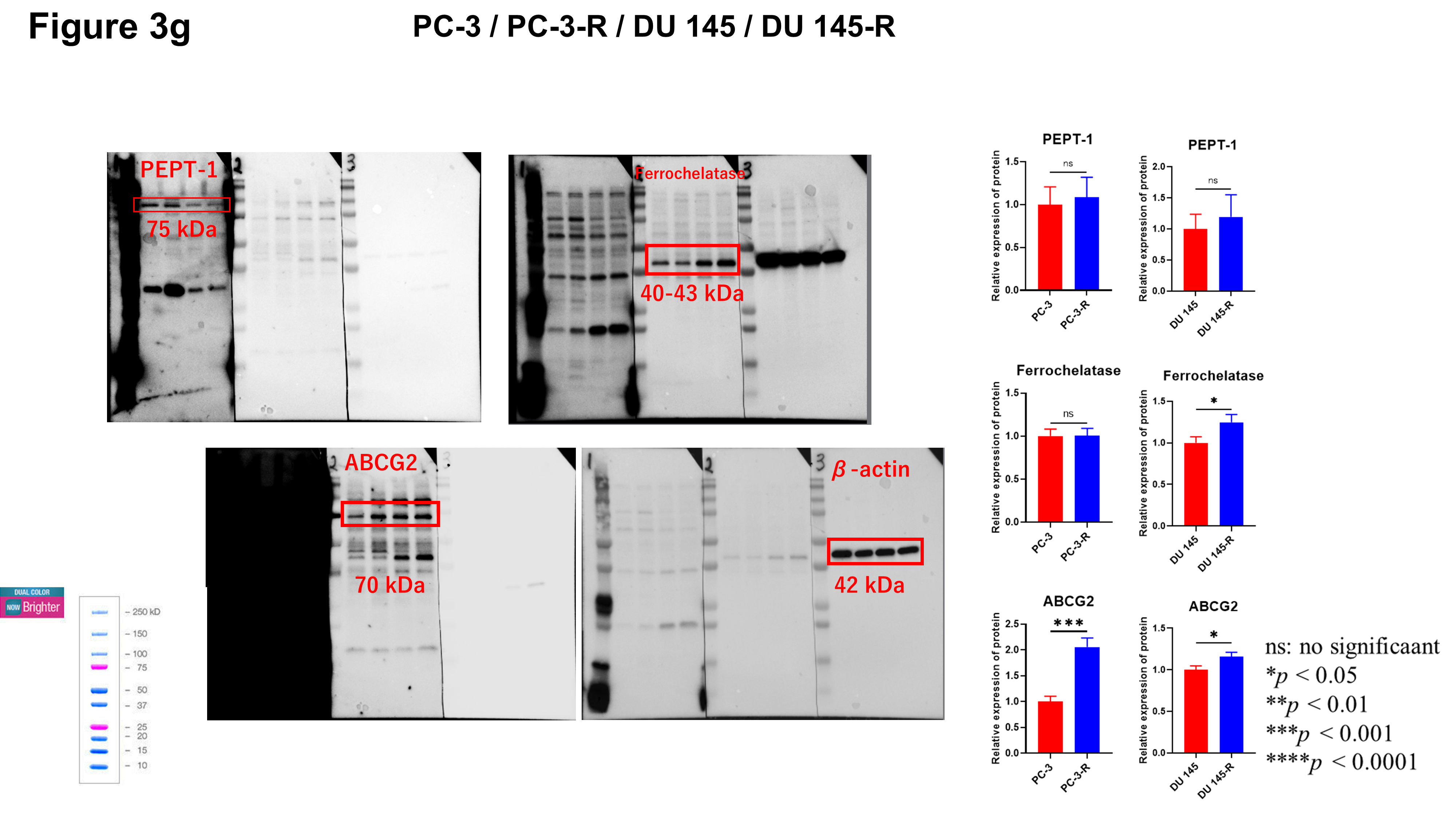

Supplement: Supplementary file 1 [file cancers-17-01286-s001.zip › cancers-3536058-File S1/Figure 3g.TIF]

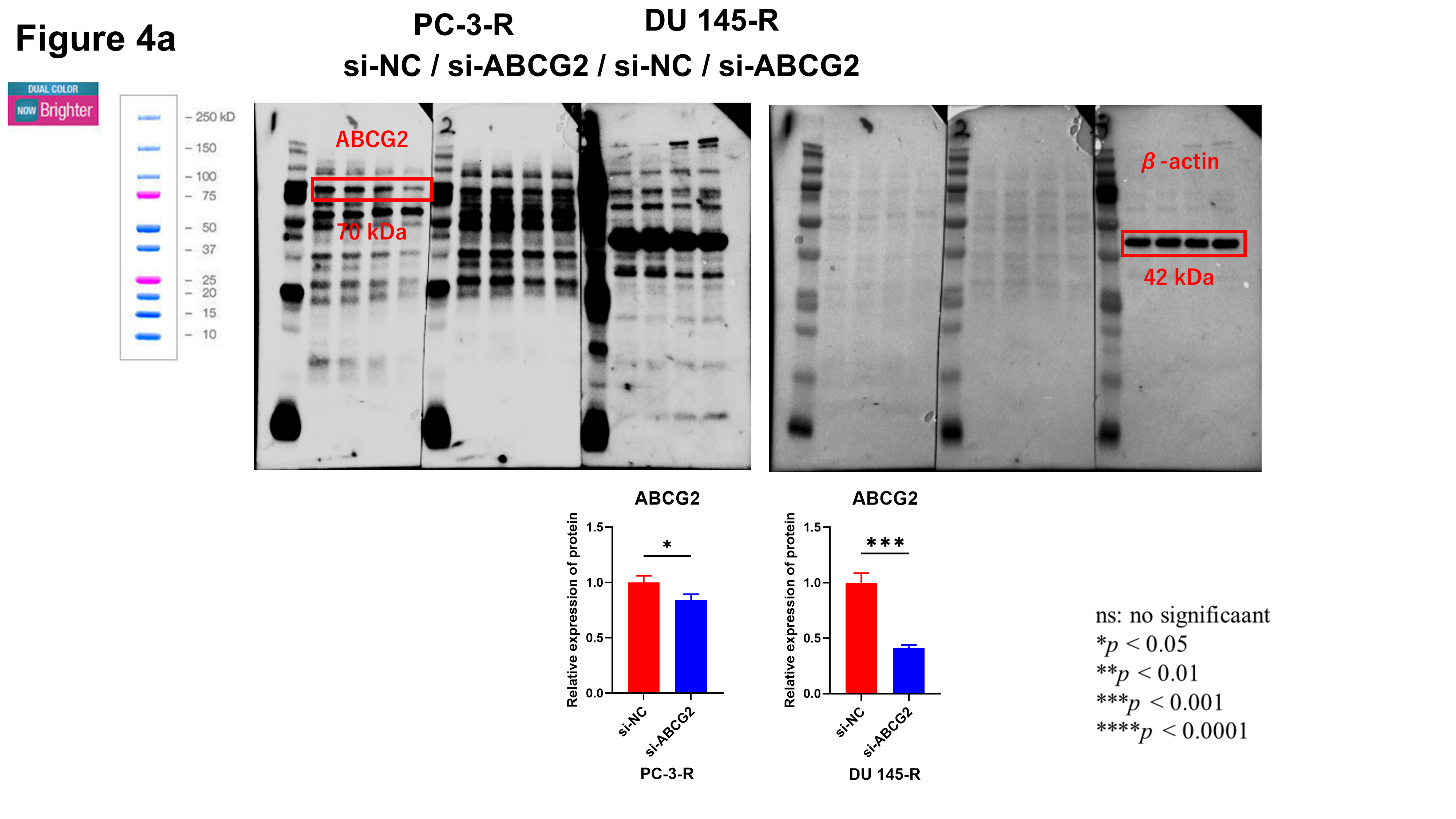

Supplement: Supplementary file 1 [file cancers-17-01286-s001.zip › cancers-3536058-File S1/Figure 4a.TIF]

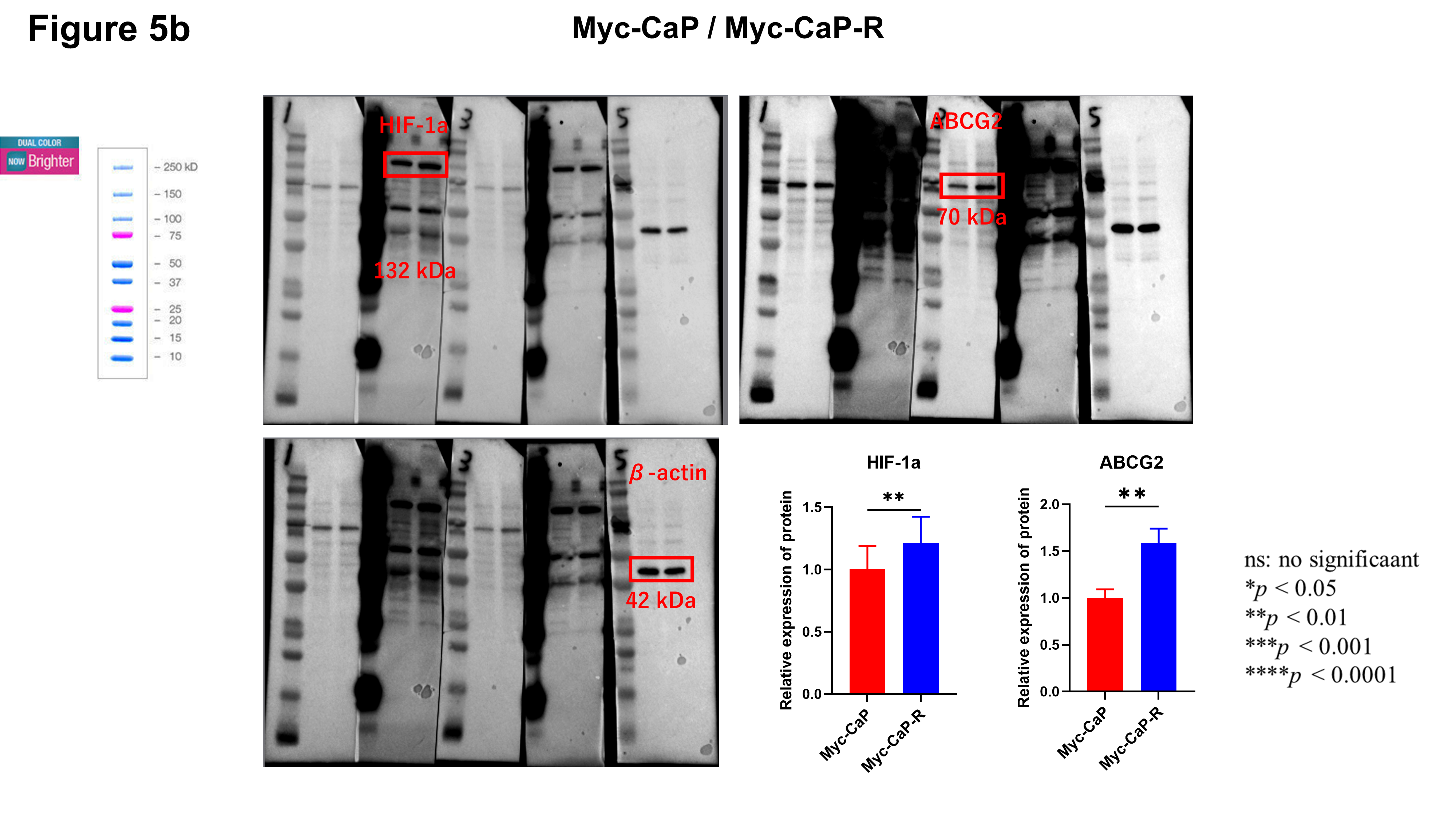

Supplement: Supplementary file 1 [file cancers-17-01286-s001.zip › cancers-3536058-File S1/Figure 5b.TIF]

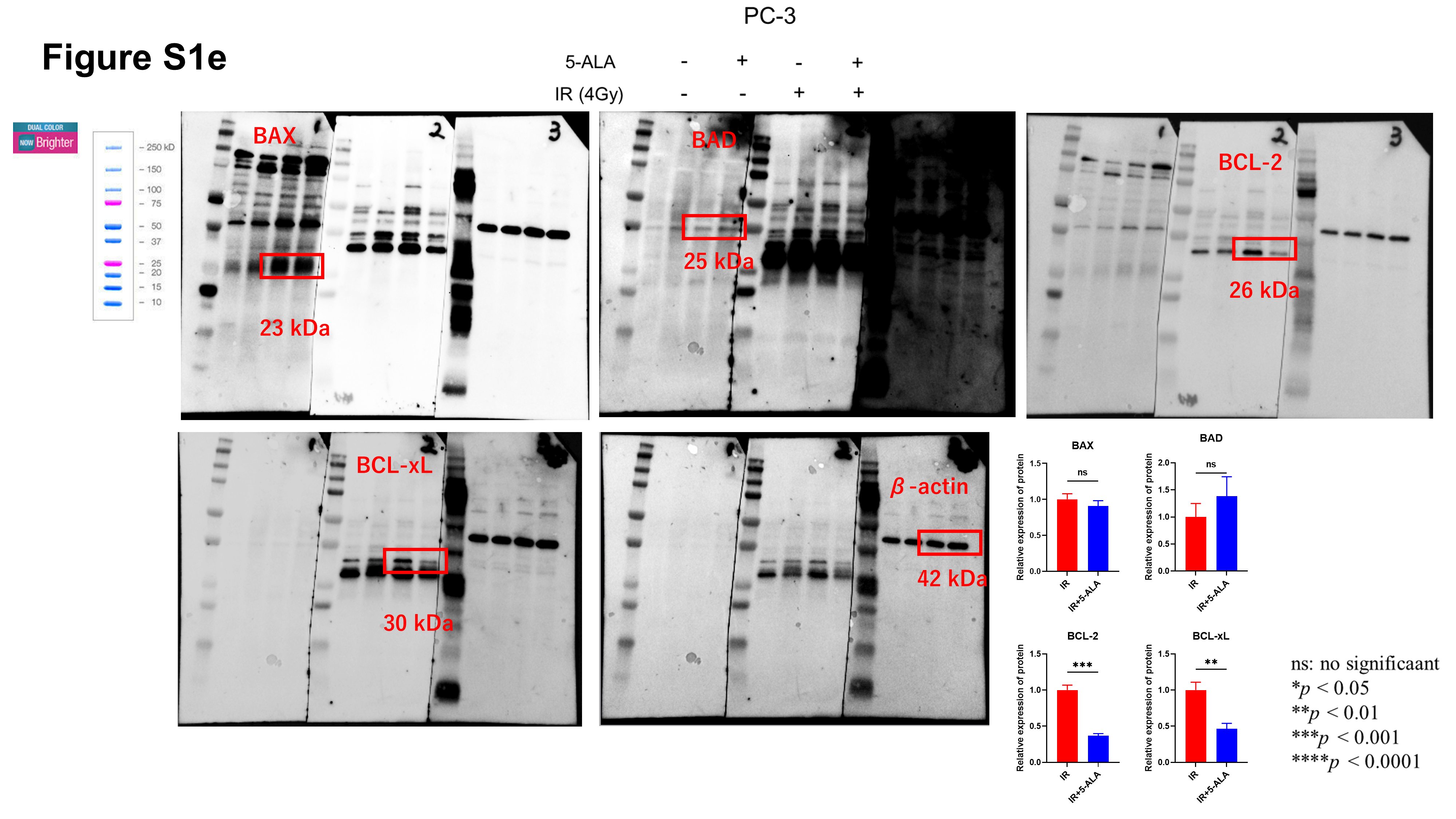

Supplement: Supplementary file 1 [file cancers-17-01286-s001.zip › cancers-3536058-File S1/Figure S1e.TIF]

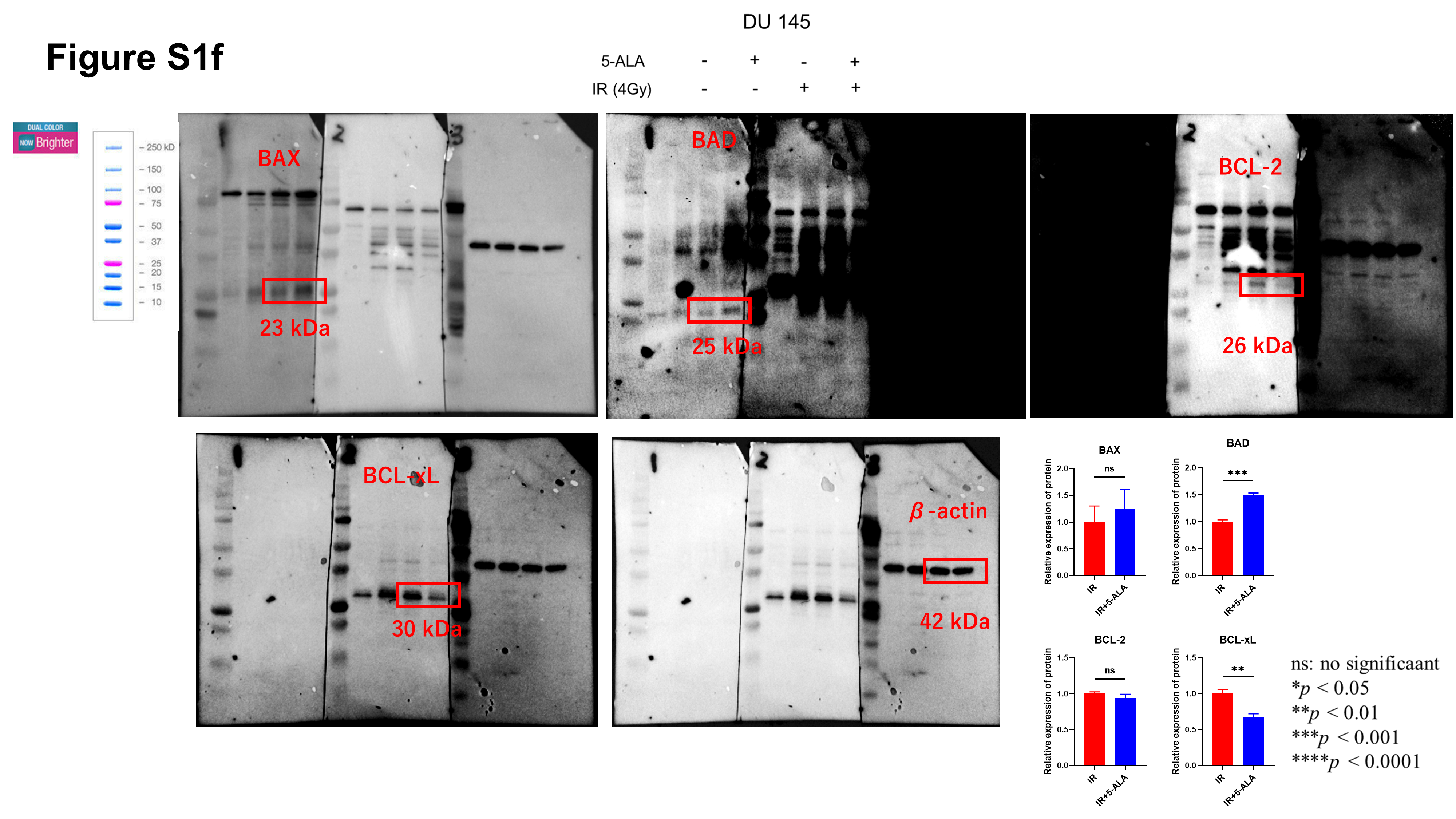

Supplement: Supplementary file 1 [file cancers-17-01286-s001.zip › cancers-3536058-File S1/Figure S1f.TIF]
